# Fuels Mediate the Influence of Climate Teleconnections on Wildfires in Dryland Ecosystems

**DOI:** 10.1111/gcb.70406

**Published:** 2025-08-06

**Authors:** Yuquan Qu, Sander Veraverbeke, Diego G. Miralles, Jingfang Fan, Harry Vereecken, Carsten Montzka

**Affiliations:** ^1^ Institute of Bio‐ and Geosciences: Agrosphere (IBG‐3) Forschungszentrum Jülich GmbH Jülich Germany; ^2^ Faculty of Science Vrije Universiteit Amsterdam Amsterdam the Netherlands; ^3^ School of Environmental Sciences University of East Anglia Norwich UK; ^4^ Hydro‐Climate Extremes Lab Ghent University Ghent Belgium; ^5^ School of Systems Science Beijing Normal University Beijing China; ^6^ Potsdam Institute for Climate Impact Research Potsdam Germany

**Keywords:** fuel and weather conditions, hot spot, mediating effect, teleconnection, time lag, wildfire

## Abstract

Climate teleconnections modulate regional wildfire occurrence. Understanding the underlying mechanisms is critical for sub‐seasonal to annual wildfire predictions since the magnitude of certain teleconnection climate modes (TCMs) intensifies or they may undergo phase shifts. Here, we study how TCMs govern wildfire activity and compare the effects of weather and fuels in mediating the influence of TCMs on wildfires. Globally, burned area (BA) is predictable by a single TCM in 25.4% of the burnable (vegetated) regions, with Australia and eastern Siberia identified as the two hot spots with the highest probability out of a total of 10. Tropical oceans are the primary sources of teleconnection‐driven variability in global BA. Our study finds that in dryland hot spots such as Australia, the Horn of Africa, and the northern Middle East, the lagged mediating effects of fuels outweigh the immediate mediating effects of weather. Whereas in hot spots with dense vegetation, like northeastern South America and Southeast Asia, the immediate mediating effects of weather are generally more dominant. In other hot spots, fuels can still serve as a key pathway through which specific TCMs influence wildfire activity. This study highlights the important role of fuels in transmitting the delayed impacts of TCMs‐induced weather anomalies on regional wildfire activity. This study also underlines the importance of refining fuel management strategies and integrating fuel conditions in teleconnection‐related wildfire attribution and prediction frameworks, which is crucial given the projected changing patterns of teleconnections.

## Graphical abstract

Climate teleconnections, such as those driven by El Niño–Southern Oscillation (ENSO), modulate regional wildfire activity via the immediate mediating effect of weather and the delayed mediating effect of fuel. We show that in drylands such as Australia, the Horn of Africa, and the northern Middle East, the fuel‐related lagged effect plays a stronger role than the immediate weather effect. This study underscores the importance of integrating fuel conditions into wildfire attribution and prediction frameworks.

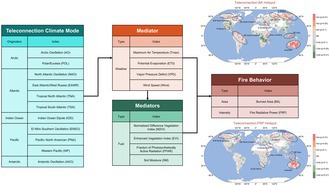

## 1 Introduction

Wildfires are projected to increase in regions and seasons that are not usually regarded as fire‐prone due to land‐use changes and drier and warmer conditions under anthropogenic warming (Andela et al. 2017; Brown et al. 2023; Clarke et al. 2022). Drought, deforestation, and forest degradation may disrupt natural barriers, enabling wildfires in wet ecosystems (Clarke et al. 2022). In turn, wildfires intensify climate change by modulating above‐ and below‐ground carbon storage, surface albedo, greenhouse gases, and aerosols (Page et al. 2002; Randerson et al. 2006; Ward et al. 2012). Effective fuel and wildfire management is essential for conserving ecological services, as well as human health and property (Moritz et al. 2014). Climate teleconnections describe significant climate responses in a region caused by distant disturbance sources (e.g., atmospheric pressure or sea surface temperature variations) through atmospheric propagation (Cardil et al. 2023; Zhao et al. 2022). Some teleconnection climate modes (TCMs), e.g., El Niño–Southern Oscillation, modulate wildfire preconditions through immediate or lagged effects (Cardil et al. 2023; Wu et al. 2021). In southeastern Siberia, for example, the Arctic Oscillation modulates snowmelt time, and thus fuel dryness and wildfire occurrence in spring (Kim et al. 2020). Wildfire occurrence increases when high‐pressure systems resulting from TCMs persist, because the suppressed precipitation and increased temperature and solar radiation desiccate fuels (Zhao et al. 2022). TCMs can also alter the path and strength of large‐scale airflow (e.g., westerlies and jet streams), prevent vapor import, and cause droughts (Jain and Flannigan 2021). The accuracy and time window of wildfire predictions, therefore, can be improved and extended as seasonal to interannual forecasts of some TCMs are feasible (Ham et al. 2019; Liu et al. 2023; Lu et al. 2022).

Predicting the fate of TCMs under climate change can help project future wildfire occurrences. An increase in the magnitude of El Niño–Southern Oscillation (Cai et al. 2023, 2022) and its resulting variability of the Tropical North Atlantic pattern (Yang et al. 2021) and the Pacific–North American pattern (Cai et al. 2021) under anthropogenic warming is projected. In southeastern Australia, El Niño usually coincides with heatwaves, droughts, and conducive fire weather (Abram et al. 2021). In the drylands of Australia, La Niña brings more precipitation, and thus more fuels accumulate, potentially increasing the risk of wildfires (AFAC 2023; Zhang 2023). Meanwhile, the frequency of strong/multi‐year La Niña events may increase as they usually follow strong El Niño events to balance the large heat budget (Cai et al. 2023; Geng et al. 2023). This projected increasing magnitude of El Niño–Southern Oscillation, therefore, may pose challenges to humanity in managing extreme wildfires. Moreover, due to the asymmetrical and faster warming of the eastern tropical Pacific, even a mild El Niño can cause considerable atmospheric convection and result in significant climate impacts (Cai et al. 2021). In addition to magnitude changes, some TCMs may offset the effects of anthropogenic warming or undergo phase shifts that need to be considered in future wildfire predictions. For example, wildfire emissions in South America are decreasing due to the weakening of the positive phase of the Atlantic Multidecadal Oscillation. However, if anthropogenic warming continues to intensify, this decreasing trend may be overturned (Wang and Huang 2022). In recent decades, the Antarctic Oscillation has tended to be in its positive phase because of anthropogenic warming and stratospheric ozone depletion (Arblaster and Meehl 2006; Thompson et al. 2011). However, the Montreal Protocol successfully stabilized the Antarctic ozone depletion, weakening the positive tendency of the Antarctic Oscillation (Banerjee et al. 2020), which may exacerbate wildfires in Australia in the future (Abram et al. 2021).

Here, we explore the variables that mediate the relationships between TCMs and wildfires, considering both weather (top‐down) and fuels (bottom‐up) as mediators. The relationships between TCMs and wildfires, as well as the corresponding physical mechanisms, have been studied regionally and globally (Cardil et al. 2023; Chen, Morton, et al. 2016; Le et al. 2022; Zhao et al. 2022). However, these studies did not provide a comprehensive analysis of the underlying pathways or focused solely on the weather variabilities induced by TCMs and did not account for the role of fuels, which also play an important role in wildfire activities (Qu et al. 2023). The pathways between TCMs and wildfires, that is, via weather or fuel mediators, are yet unclear. In this study, we aim to answer three main questions: (a) where are the hot spots in which wildfires are highly predictable by TCMs? (b) how do TCMs influence global and regional wildfires? (c) what are the dominant mediators between TCMs and wildfires, weather or fuels? We underline the important role of fuel mediators in linking TCMs and wildfires, especially in dryland hot spots, and provide a unique pathway analysis framework to disentangle the influence of TCMs on wildfires from other drivers. It enables us to refine fuel management strategies, improve wildfire attribution and prediction frameworks, and better prepare for potentially high‐risk fire seasons induced by TCMs.

## 2 Materials and Methods

### 2.1 Wildfire and Mediator Data

Burned area (BA) used to characterize fire behavior was obtained from MCD64A1 v061 (Giglio et al. 2021) at 500 m spatial resolution and daily temporal resolution. Fire radiative power (FRP) complements the spatial information provided by BA by measuring fire intensity via the energy released during active burning. FRP was obtained from MYD14A1 v061 (Giglio and Justice 2021) at 1 km spatial resolution and daily temporal resolution. Additional information regarding the processing of FRP is available in the Supporting Information. The mediators used were classified as weather (top‐down) and fuel (bottom‐up) mediators based on a previous study (Qu et al. 2023). Weather mediators include the maximum 2 m air temperature (Tmax), potential evaporation (ET0), vapor pressure deficit (VPD), and 10 m wind speed (Wind). The fuel mediators include normalized difference vegetation index (NDVI), enhanced vegetation index (EVI), fraction of photosynthetically active radiation (FPAR), and 0–7 cm soil moisture (SM). Tmax, ET0, Wind, and SM were acquired from ECMWF Reanalysis v5 (ERA5) monthly averaged data on single levels (Hersbach et al. 2023) with a spatial resolution of 0.25°. VPD was calculated based on the minimum and maximum 2 m air temperatures and dewpoint temperature from ERA5. The calculation of VPD can be found in the Supporting Information. NDVI and EVI were obtained from monthly MOD13C2 v061 (Didan 2021) with a spatial resolution of 0.05°. FPAR was acquired from MCD15A3H v061 (Myneni et al. 2021) with a spatial resolution of 500 m and a temporal resolution of 4 days. All the datasets span from 2003 to 2024. They were resampled to 1° spatial resolution and monthly temporal resolution by averaging (for the mediators), aggregating (for BA), and calculating the 95th percentile (for FRP). For simplicity, we used BA as an example to illustrate the methodology in the following part of this section.

### 2.2 Teleconnection Climate Mode Data

In this study, 11 teleconnection climate modes (TCMs) were involved, including:

Arctic Oscillation (AO): AO, also known as the Northern Annular Mode (NAM), is characterized by the sea‐level atmospheric pressure difference between the Arctic and Northern Hemisphere mid‐latitudes (37°–45° N), describing the north–south shift of the storm‐steering and mid‐latitude jet stream (Thompson and Wallace 1998).

Polar/Eurasia pattern (POL): POL describes the changes in the strength of the atmospheric circulation centered in the north pole and corresponding changes in the midlatitude circulation over Eurasia (Barnston and Livezey 1987).

North Atlantic Oscillation (NAO): NAO is characterized by the sea‐level atmospheric pressure difference between the Icelandic Low and the Azores High, influencing the strength of the Atlantic jet stream and the location of the storm track (Jones et al. 1997).

East Atlantic/West Russia Pattern (EAWR): EAWR describes a dipole of atmospheric pressure over western Europe and western Russia, with four pressure anomaly centers over western Europe, northern China, northern Atlantic, and northern Caspian Sea (Barnston and Livezey 1987).

Tropical North Atlantic pattern (TNA): TNA is defined as the average sea surface temperature anomalies in regions located in 5.5°–23.5° N and 15°–57.5° W, influencing the north–south shift of the Intertropical Convergence Zone (ITCZ) and strength of northeasterly trade winds (Enfield et al. 1999).

Tropical South Atlantic pattern (TSA): Like the TNA but for the regions located in 30° W–10° E and 0°–20° S (Enfield et al. 1999).

Indian Ocean Dipole (IOD): IOD describes the sea surface temperature difference between the western Indian Ocean (50°–70° E, 10° S–10° N) and the southeastern Indian Ocean (90°–110° E, 0°–10° S) (Saji et al. 1999).

El Niño Southern Oscillation (ENSO): ENSO is one of the most important natural drivers of global climate variability, describing the oscillating warm and cold sea surface temperature anomalies in the central and eastern tropical Pacific (McPhaden et al. 2006). The Niño 3.4 sea surface temperature index, which is defined by the average sea surface temperature anomalies in regions located in 5° S–5° N and 120°–170° W, was used.

Pacific–North American pattern (PNA): PNA is characterized by the atmospheric pressure difference between the North Pacific and the North American continent, influencing the strength and location of the East Asian jet stream (Barnston and Livezey 1987). Four atmospheric pressure anomaly centers are located around Hawaii, the mountain region of western North America, south of Alaska, and the southeastern United States.

Western Pacific pattern (WP): WP is characterized by the dipole atmospheric pressure pattern over the North Pacific, with one anomaly center over the Kamchatka Peninsula and another anomaly center over southeastern Asia and the western subtropical North Pacific, describing the intensity and meridional shifts of the East Asian jet stream (Mo and Livezey 1986; Wallace and Gutzler 1981).

Antarctic Oscillation (AAO): AAO, also known as the Southern Annular Mode (SAM), is characterized by the sea‐level atmospheric pressure difference between the Antarctic regions and the Southern Hemisphere mid‐latitudes (40°–50° S), describing the north–south shift of the Southern Westerlies (Gong and Wang 1999).

The indices for these TCMs were obtained from the Koninklijk Nederlands Meteorologisch Instituut (KNMI) Climate Explorer (http://climexp.knmi.nl/) and the National Oceanic and Atmospheric Administration Physical Sciences Laboratory (NOAA PSL, https://psl.noaa.gov/), spanning from 2003 to 2024 at a monthly temporal resolution.

### 2.3 Time Series Processing

To study the influence of TCMs on BA, we first determined the fire season peak in each 1° grid cell to avoid the influence of the weak correlations between TCMs and non‐fire season low BA. The fire season peak was defined as the three consecutive months with the highest accumulated BA, which was aligned with a previous study (Cardil et al. 2023). To detect the immediate and lagged effects of TCMs on BA (Cardil et al. 2023) and corresponding pathways (TCMs→mediators→BA), we introduced time lags into TCMs and mediators by switching their time series 0–24 steps (months) backward. The maximum time lag was set as 24 months, considering that the fuel buildup process may take 1–2 years (Qu et al. 2023). Since we focus on the influence of TCMs and mediators on BA, a wildfire event can only coincide with or follow (but not precede) TCMs and mediators, reflecting the causality direction. Subsequent regressions and correlation analyses were conducted based on the fire season peak BA and time‐lagged TCMs and mediators. To minimize the influence of long‐term trends and seasonal cycles, we first removed linear trends from TCMs, mediators, and BA over the entire period using linear regression. We then calculated anomalies in mediators and BA by subtracting the long‐term means of the corresponding month.

### 2.4 Partial Least Squares Regression

Partial least squares regression (PLSR) was applied to each grid cell globally with time‐lagged (0–24 months) TCM as the independent variables and BA as the dependent variable. PLSR brings the advantages of principal component analysis to ordinary least squares regression. Instead of applying regression on the original variables, PLSR projects the independent and dependent variables to a smaller set of uncorrelated components that account for as much covariance as possible. The corresponding equations are presented in Equations (1) and (2).

(1) $$X_{j}=\sum_{t=0}^{24}{\mathrm{TCM}}_{t}\times w_{t}^{j}$$

(2) $$\mathrm{BA}=\sum_{j=1}^{k}c_{j}\times X_{j}+\varepsilon$$

where $X_{j}$ is the $j^{\mathrm{th}}$ projected component, ${\mathrm{TCM}}_{t}$ is the TCM with a time lag of t (ranging from 0 to 24), $w_{t}^{j}$ is the weight of ${\mathrm{TCM}}_{t}$, $c_{j}$ is the regression coefficient for $X_{j}$, and ε is the residual. Note that both the $w_{t}^{j}$ and $c_{j}$ are estimated simultaneously to maximize the covariance between the projected components and BA.

Because the regression is based on these projected components rather than the original variables, compared with other regression methods such as Lasso or Ridge regression, which are more suitable for causal inference, traditional statistical tests (e.g., the significance of individual coefficients) become less meaningful for PLSR. Nevertheless, the overall performance of the PLSR model can still be effectively evaluated. In this study, we assessed its significance through 10‐fold cross‐validation and by estimating the *p*‐value of the *F*‐statistic. To ensure the robustness of the model, we further validated it using a separate test set, with 70% of the data allocated for training and 30% for testing. In each grid cell, the BA predictability of an individual TCM was expressed as the coefficient of determination (*R* ^2^) between predicted and observed BA from the test set. BA was considered predictable when the *F*‐statistic of the PLSR model indicated significance at the 0.05 level. More details about why PLSR was chosen can be found in the Supporting Information.

### 2.5 Hot Spot Analysis

A hot spot is defined as a geographically clustered group of grid cells where the TCM exhibits higher‐than‐average BA predictability. A hot spot analysis tool in ArcGIS (Spatial Statistics Tools/Mapping Clusters/Hot Spot Analysis), based on the Getis‐Ord Gi* statistic (Getis and Ord 1992; Ord and Getis 1995), was used to identify hot/cold spots from the BA predictability global pattern. We applied hot spot analysis to the global pattern of maximum BA predictability, defined as the highest BA predictability from TCMs at each grid cell. We also applied the same hot spot analysis to the BA predictability of each TCM to determine their respective hot spots. More details about the Getis‐Ord Gi* statistic hot spot analysis can be found in the Supporting Information.

### 2.6 Time Lag Analysis

For all the grid cells within an identified hot spot, the Pearson correlation coefficients between TCMs and BA were calculated over a specific time lag range (0–24 months). If the correlation was not statistically significant (*p* > 0.05), the correlation coefficient was set to zero. The time lag distribution of a specific TCM within a hot spot was then calculated by averaging the absolute values of the correlation coefficients from all grid cells within that hot spot and across all time lags from 0 to 24 months. To facilitate the comparison across different TCMs and hot spots, we normalized all time lag distributions to a 0–1 scale. The positive/negative fraction in each time lag is calculated by dividing the sum of the absolute values of positive/negative correlation coefficients by the sum of the absolute values of all correlation coefficients from that time lag.

### 2.7 Pathway Analysis

A three‐step ordinary least squares regression procedure was used to calculate the contribution of a TCM to BA through a specific mediator. In the first regression (Equation 3), we input a time‐lagged TCM (${\mathrm{TCM}}_{x,i+j}$) as the independent variable and BA as the dependent variable. We recorded the coefficient of determination between predicted and observed BA as well as the *p*‐value of the *F*‐statistic as $R_{1}^{2}$ and $p_{1}$. We only proceeded with the next two regressions if the first regression was statistically significant ($p_{1}$ < 0.05).

(3) $$\mathrm{BA}=a_{1}+b_{1}\times {\mathrm{TCM}}_{x,i+j}+c_{1}$$

where x is the TCM index, i is the time lag between TCM and mediator, j is the time lag between mediator and BA, i + j is the time lag between TCM and BA, $a_{1}$ is the intercept, $b_{1}$ is the regression coefficient, and $c_{1}$ is the residual. Note that i and j range from 0 to 24, and i + j should be no more than 24.

In the second regression (Equation 4), we input the same TCM with the same time lag (${\mathrm{TCM}}_{x,i+j}$) as the independent variable and a time‐lagged mediator (${\text{Mediator}}_{y,j}$) as the dependent variable. We recorded the coefficient of determination between the predicted and observed mediator as well as the *p*‐value of the *F*‐statistic as $R_{2}^{2}$ and $p_{2}$.

(4) $${\text{Mediator}}_{y,j}=a_{2}+b_{2}\times {\mathrm{TCM}}_{x,i+j}+c_{2}$$

where y is the mediator index, $a_{2}$ is the intercept, $b_{2}$ is the regression coefficient, and $c_{2}$ is the residual.

In the third regression (Equation 5), we input the same mediator with the same time lag (${\text{Mediator}}_{y,j}$) as the independent variable and BA as the dependent variable. We recorded the coefficient of determination between the predicted and observed BA as well as the *p*‐value of the *F*‐statistic as $R_{3}^{2}$ and $p_{3}$.

(5) $$\mathrm{BA}=a_{3}+b_{3}\times {\text{Mediator}}_{y,j}+c_{3}$$

where $a_{3}$ is the intercept, $b_{3}$ is the regression coefficient, and $c_{3}$ is the residual error.

Then, the pathway contribution of a TCM (${\mathrm{TCM}}_{x,i+j}$) to BA through a mediator (${\text{Mediator}}_{y,j}$) can be calculated from Equation (6) if all $p_{1}$, $p_{2}$, and $p_{3}$ are less than 0.05, otherwise, the contribution is set as zero.

(6) $$\text{Contribution}=R_{1}^{2}\times R_{2}^{2}$$

In each hot spot, we summed all contributions from the same TCM through the same mediator and all possible time lags in all grid cells. Then, the summed contributions were compared to find the dominant mediator and mediator group in that hot spot. The justification and assumptions of the pathway analysis can be found in the Supporting Information. A detailed flowchart of the pathway contribution calculation is provided in Figure S1.

## 3 Results

### 3.1 Climate Teleconnection–Wildfire Hot Spots

**FIGURE 1:**
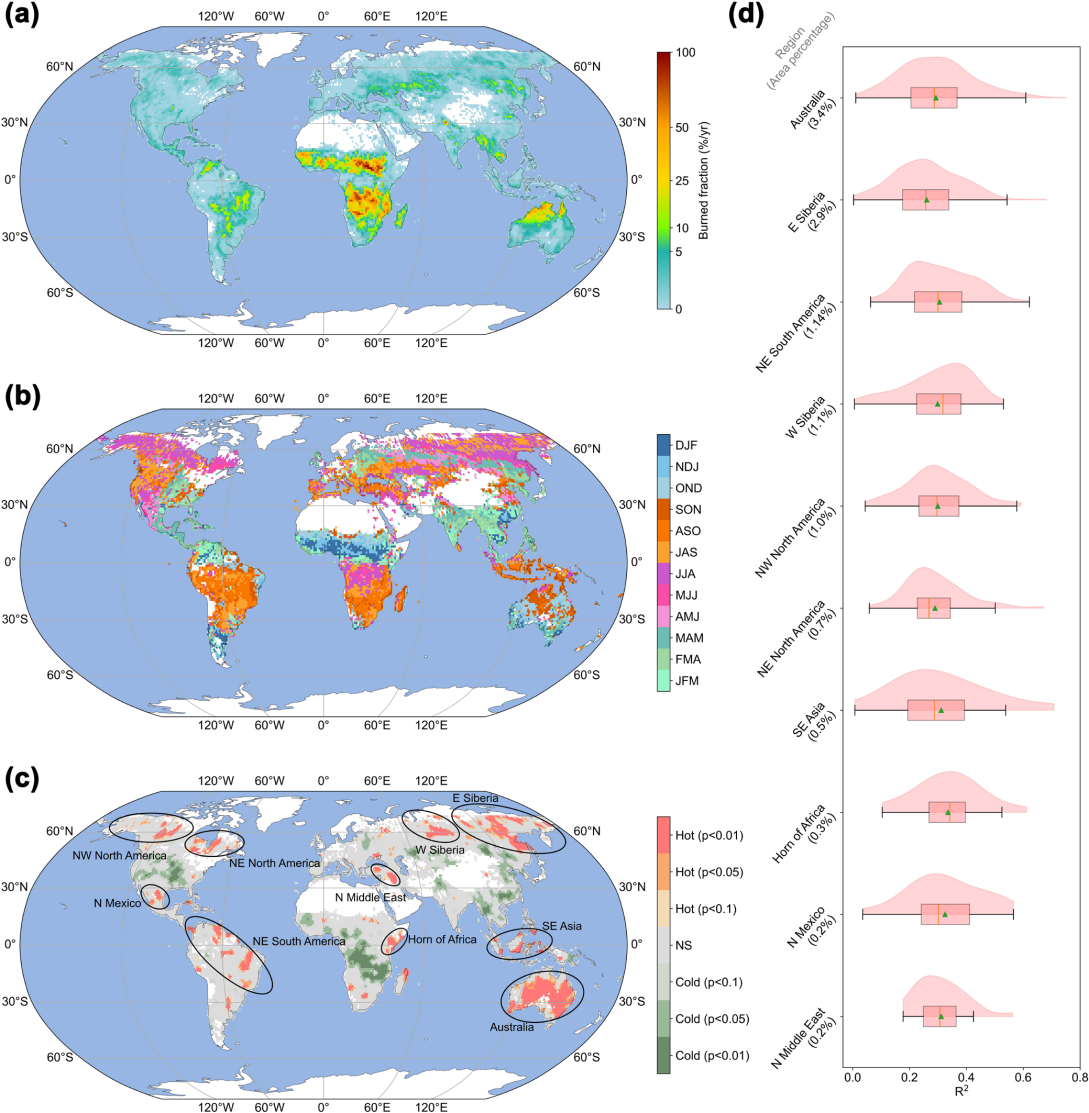
Global burned area (BA) pattern and hot spots where BA is highly predictable by teleconnection climate modes (TCMs). (a) Spatial pattern of the mean annual BA fraction in each 1° grid cell over 2003–2024. (b) Spatial pattern of fire season peak. The fire season peak was defined as the three consecutive months with the highest accumulated BA. The capital letters in the legend are the combination of the initial letters of the three consecutive months. (c) Hot/cold spots where TCMs show higher/lower BA predictability. They are clusters of grid cells that show higher/lower local BA predictability than the global grid cells. The *p*‐values indicate whether the difference between local clusters and global grid cells is statistically significant. Hot/cold spots are labeled as Hot/Cold with *p*‐values in the legend. The black ellipses mark hot spots with a significance level of 0.01, including northwestern North America, northeastern North America, northern Mexico, northeastern South America, the Horn of Africa, northern Middle East, western Siberia, eastern Siberia, Southeast Asia, and Australia. The gray regions (not significant, NS, *p* > 0.1) are neither hot spots nor cold spots. (d) The distribution of *R* ^2^ between predicted and observed BA in each hot spot. The boxplot shows the minimum, first quartile, median (red lines), mean (green triangles), third quartile, and maximum. The violin plot shows the probability density of *R* ^2^. The hot spots were ranked based on the proportion of their area relative to the total global burnable area (values shown in parentheses).

Ten hot spots where TCMs show higher‐than‐average BA predictability were determined, including northwestern North America, northeastern North America, northern Mexico, northeastern South America, the Horn of Africa, northern Middle East, western Siberia, eastern Siberia, Southeast Asia, and Australia (Figure 1c). Australia and eastern Siberia were two large hot spots, accounting for 3.4% and 2.9% of the global burnable (vegetated) area (Figure 1d). None of the tropical savannas with high BA fractions were determined as hot spots (Figure 1a,c), probably due to their consistent dry season and flammable fine fuels (Chen, Morton, et al. 2016). Several cold spots where TCMs show lower‐than‐average BA predictability were also determined, including the Central United States, the southern equatorial region of sub‐Saharan Africa, northern and southeastern China, and northwestern Indochina.

**FIGURE 2:**
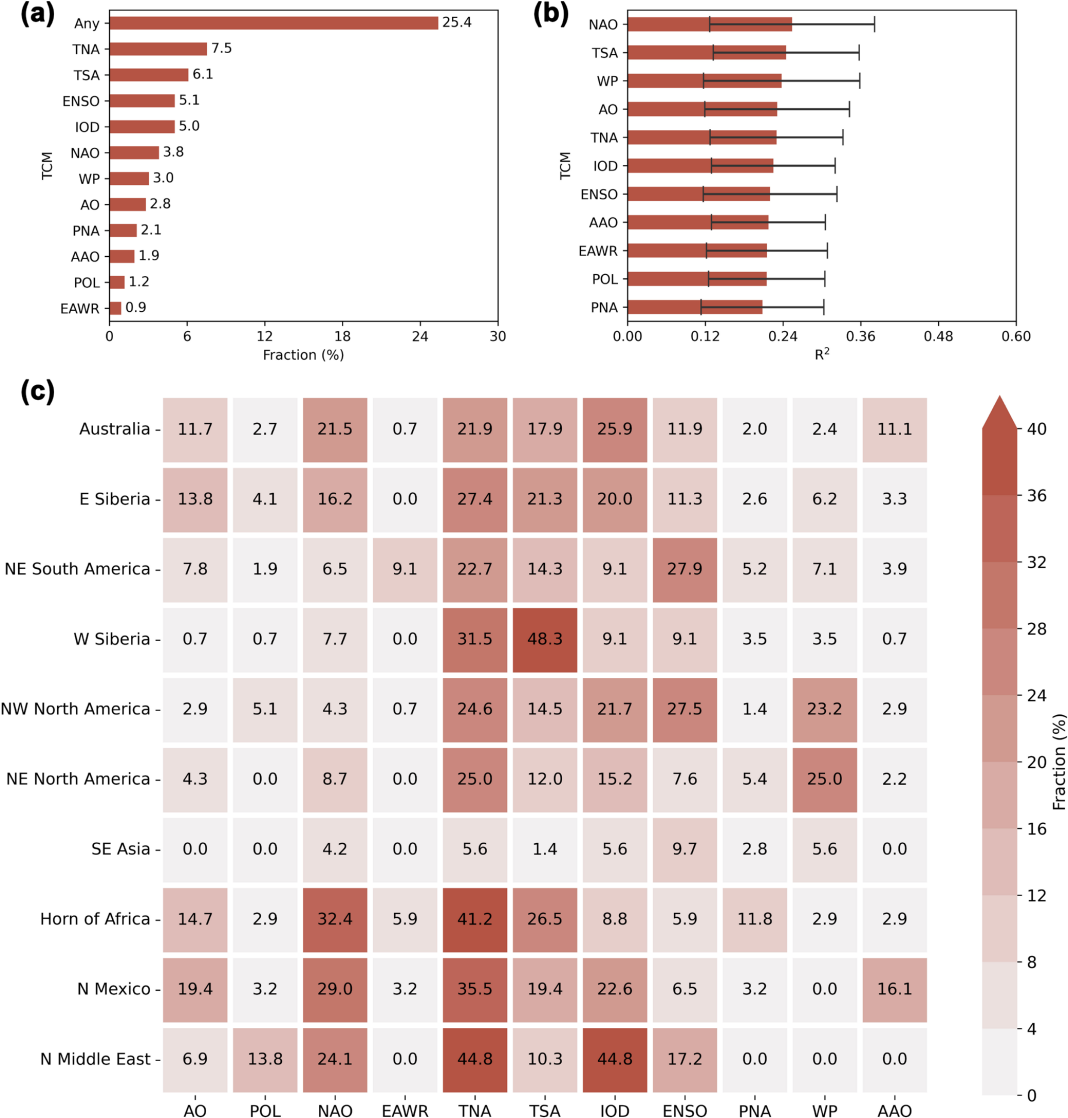
Importance of teleconnection climate modes (TCMs) in predicting burned area (BA). (a) Importance of TCMs according to the affected area fraction. The fraction was derived by dividing the number of grid cells in which BA is significantly predictable (*F*‐statistic, *p* < 0.05) by the total number of burnable grid cells globally. The label ‘Any’ indicates BA is predictable by any single TCM. The TCMs were ranked by their affected area fraction. (b) Importance of TCMs according to the global mean BA predictability. In each grid cell, the BA predictability of an individual TCM was expressed as the *R* ^2^ between predicted and observed BA. Only the grid cells where BA is significantly predictable were taken into account. The error bars show the standard deviations. The TCMs were ranked by their global mean BA predictability. (c) Importance of TCMs in hot spots according to their affected area fraction. Arctic Oscillation (AO), Polar/Eurasia pattern (POL), North Atlantic Oscillation (NAO), East Atlantic/West Russia Pattern (EAWR), Tropical North Atlantic pattern (TNA), Tropical South Atlantic pattern (TSA), Indian Ocean Dipole (IOD), El Niño Southern Oscillation (ENSO), Pacific–North American pattern (PNA), Western Pacific pattern (WP), Antarctic Oscillation (AAO).

BA in about 25.4% of global burnable areas was predictable by a single TCM (Figure 2a). TNA, TSA, ENSO, and IOD, representing the tropical Atlantic, tropical Pacific, and tropical Indian Ocean, were the most important ones according to the affected area fraction. The dominance of TNA was shown in eastern Siberia, northeastern North America, the Horn of Africa, northern Mexico, and the northern Middle East, accounting for 27.4%, 25.0%, 41.2%, 35.5%, and 44.8% of the burnable area, respectively (Figure 2c). TSA dominated in western Siberia, accounting for 48.3% of the burnable area there. ENSO showed dominance in northeastern South America (27.9%), northwestern North America (27.5%), and Southeast Asia (9.7%). IOD dominated in Australia (25.9%) and the northern Middle East (44.8%).

**FIGURE 3:**
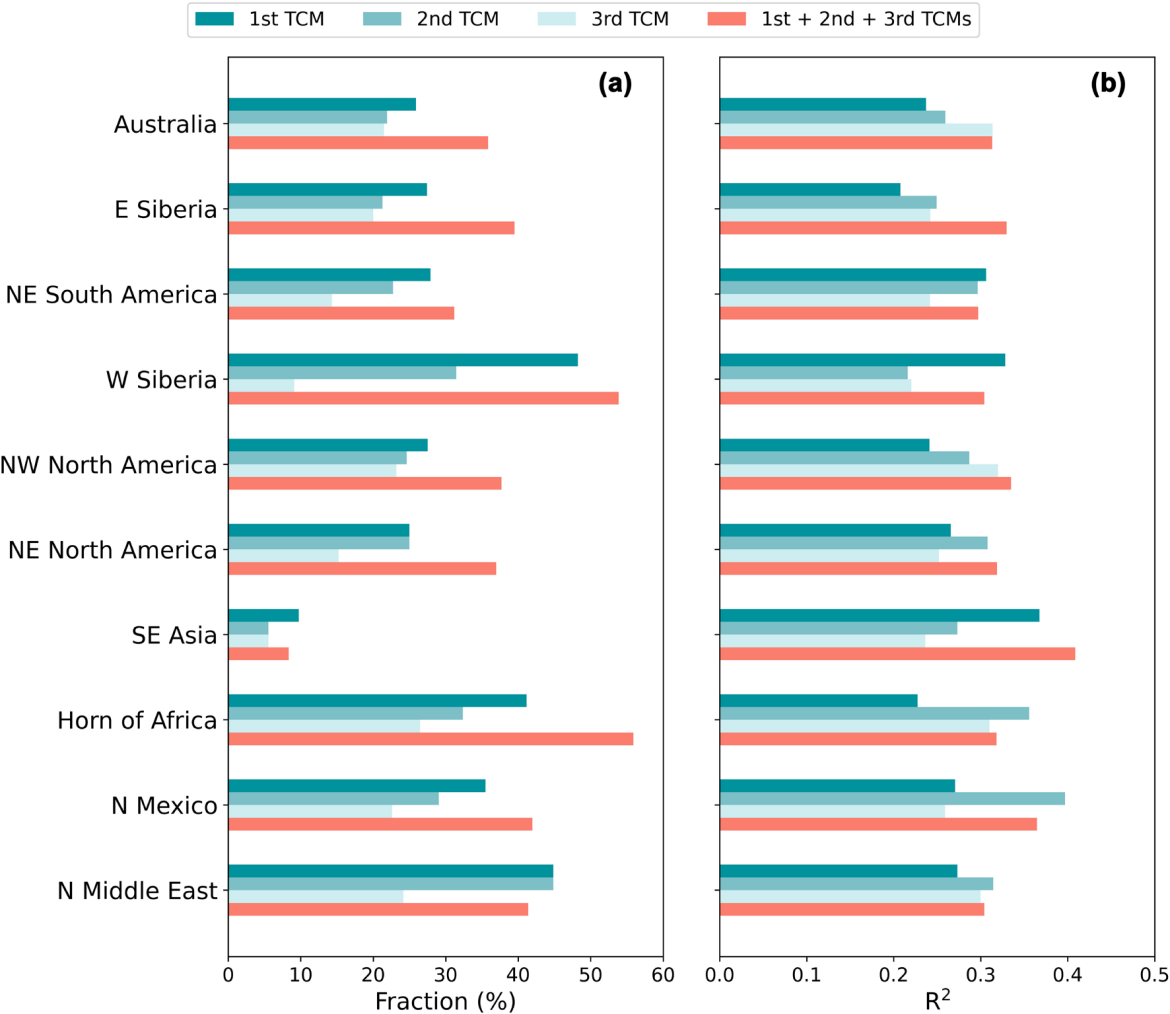
Burned area (BA) predictability in hot spots driven by teleconnection climate modes (TCMs). (a) Predictable area fraction driven by the first (1st), second (2nd), and third (3rd) dominant TCM, as well as all three TCMs (1st + 2nd + 3rd). BA was considered statistically predictable when the *F*‐statistic indicated significance at the 0.05 level. The dominance of TCM (1st, 2nd, or 3rd) in each hot spot was assessed according to the fraction of the area affected by that TCM within the region, as shown in Figure 2c. (b) BA predictability driven by the first (1st), second (2nd), and third (3rd) dominant TCM, as well as all three TCMs (1st + 2nd + 3rd). The BA predictability was expressed as the *R* ^2^ between predicted and observed BA. Only the grid cells where BA is statistically predictable were taken into account.

The BA prediction performance improved when the top three most important TCMs were combined to predict BA (3‐TCM model), in terms of both the predictable area fraction and predictability (Figure 3). For many of the hot spots, the predictable area fraction obtained from the 3‐TCM model can reach or exceed 40%. The highest fractions were observed in the Horn of Africa and western Siberia, with predictable area fractions of 55.9% and 53.8%, respectively. While Southeast Asia exhibited the lowest predictable area fraction (8.3%). The 3‐TCM model explained approximately 30%–40% of the variance in BA. Compared to the 1‐TCM model, the greatest improvement in predictability was found in eastern Siberia, where the BA explained variance increased from 21%–25% to 33%. We also investigated the influence of TCMs on FRP, a proxy for fire intensity. Nine hot spots were identified, largely consistent with those identified in the BA analysis, except for the absence of a hot spot in northeastern South America and the emergence of a new one in South Africa (Figure S4). FRP was found to be predictable by a single TCM in approximately 23.4% of global burnable areas (Figure S6). Among the 11 TCMs examined, TNA, WP, ENSO, TSA, and IOD emerged as the most influential, consistent with the findings from the BA analysis. The FRP prediction performance improved with the use of the 3‐TCM model. In many hot spots, the fraction of burnable areas with predictable FRP reached or exceeded 40%, with an explained variance between 30% and 40% (Figure S7).

### 3.2 Lagged Relationships Between Climate Teleconnections and Wildfires

**FIGURE 4:**
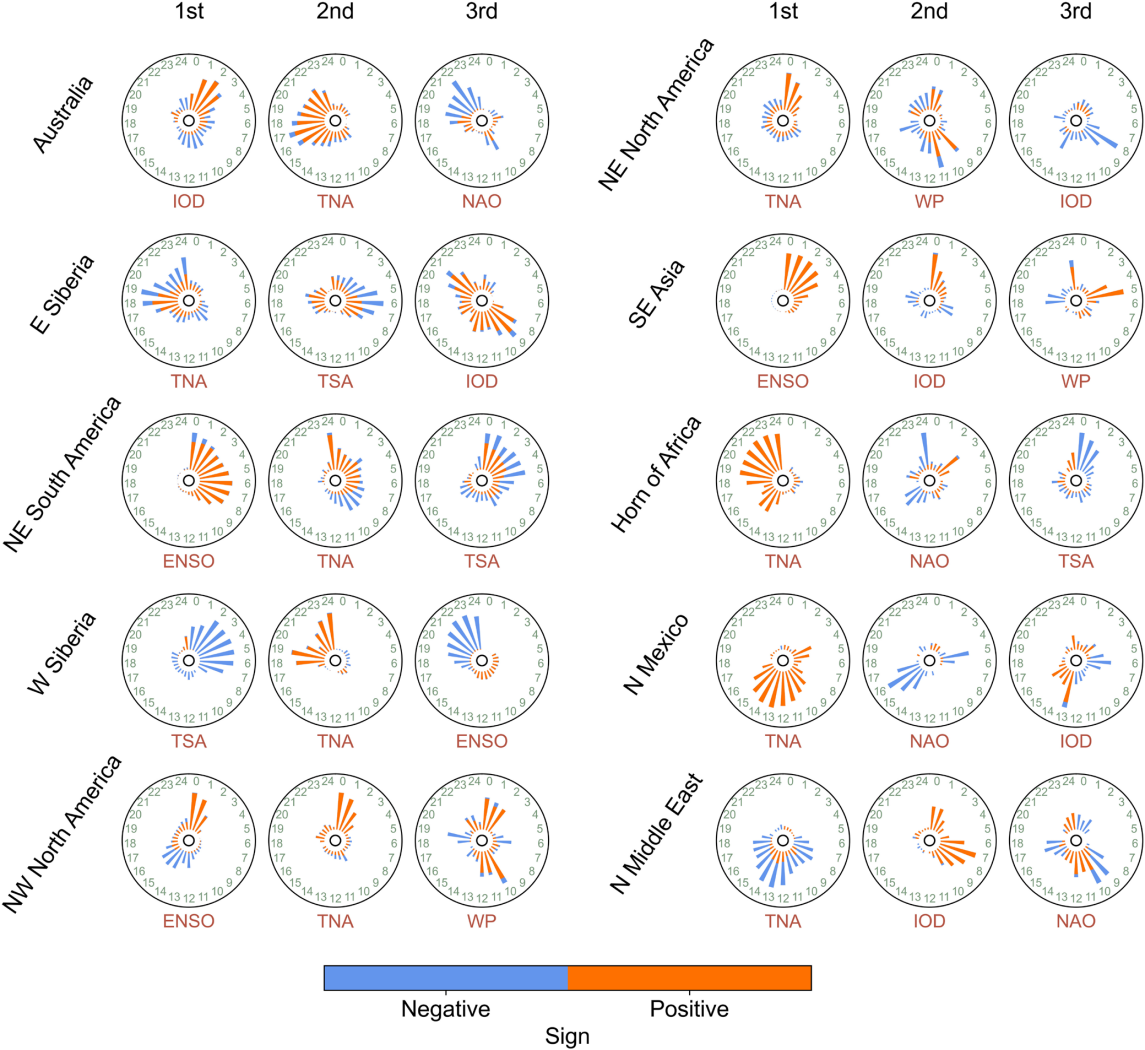
Time lags of teleconnection climate modes (TCMs). The correlation coefficients between TCMs and burned area (BA) were used to detect time lags and determine whether correlations were positive or negative. In each hot spot, the time lag distributions were calculated by summing all the absolute values of the correlation coefficients between TCMs with the same time lag (0–24 months) and BA from all grid cells within the hot spot. Only the grid cells where the correlations were significant (*p* < 0.05) were used in the summing process. For the comparison between different TCMs and hot spots, the time lag distributions were normalized to 0–1. The positive/negative fraction in each bar is calculated by dividing the sum of the absolute values of positive/negative correlation coefficients by the sum of the absolute values of all correlation coefficients. In each hot spot, only the first dominant (1st), second dominant (2nd), and third dominant (3rd) TCMs are shown according to Figure 2c.

TNA tended to show positive correlations with BA at short time lags (0–3 months) in northwestern and northeastern North America, and at longer time lags (beyond 12 months) in Australia, western Siberia, the Horn of Africa, and northern Mexico (Figure 4). Negative correlations between TNA and BA were observed in the northern Middle East at time lags of approximately 5–19 months. In northeastern South America, TNA and BA exhibited negative correlations at time lags of approximately 9 months, and positive correlations at time lags of around 0–7 and 24 months. TSA tended to show negative correlations with BA at short time lags, like eastern Siberia (around 6 months), western Siberia (0–8 months), and the Horn of Africa (0–2 months). In northeastern South America, TSA was positively correlated with BA at short time lags (0–2 months), and negatively correlated at marginally longer time lags (3–7 months). NAO was negatively correlated with BA in Australia at time lags of approximately 10 and 19–24 months, and in northern Mexico at time lags of around 5 and 16 months. In the Horn of Africa, positive correlations emerged at time lags of around 3 months, while negative correlations were observed at around 15 and 24 months. In the northern Middle East, NAO tended to show negative correlations with BA at shorter lags (less than 10 months) and positive correlations at longer lags of 10–14 and 22–24 months.

ENSO was positively correlated with BA at short time lags in northeastern South America (0–8 months) and Southeast Asia (0–4 months). Conversely, ENSO exhibited negative correlations with BA in western Siberia at time lags of 19–24 months. In northwestern North America, ENSO was positively correlated with BA at time lags of 0–2 months, whereas negative correlations emerged at time lags of approximately 14–16 months. WP tended to exhibit positive correlations with BA in northwestern North America at time lags of 0–2 and 10–12 months, and negative correlations at time lags of approximately 6 and 19 months. In northeastern North America, positive correlations were observed at time lags of 0–1 and 9–10 months, while negative correlations emerged at longer lags (beyond 12 months). In Southeast Asia, WP was positively correlated with BA at time lags of around 5 and 24 months, and negatively correlated at time lags of 18–19 months.

IOD exhibited positive correlations with BA in eastern Siberia at time lags of approximately 9 and 21 months, in Southeast Asia around 0–2 months, in northern Mexico at time lags of about 13–16 months, and in the northern Middle East at time lags of around 1 and 7 months. In contrast, negative correlations were observed in northeastern North America at time lags of around 8 and 14 months. In Australia, positive correlations appeared at short lags of approximately 0–3 months, while negative correlations emerged at longer time lags of around 10–14 months. AO, POL, PNA, and AAO were not among the top three most important TCMs in any of the defined hot spot regions. We also examined lagged relationships between TCMs and FRP (Figure S9), and the patterns revealed were largely consistent with those observed in the BA analysis.

### 3.3 Mediators Linking Climate Teleconnections and Wildfires

**FIGURE 5:**
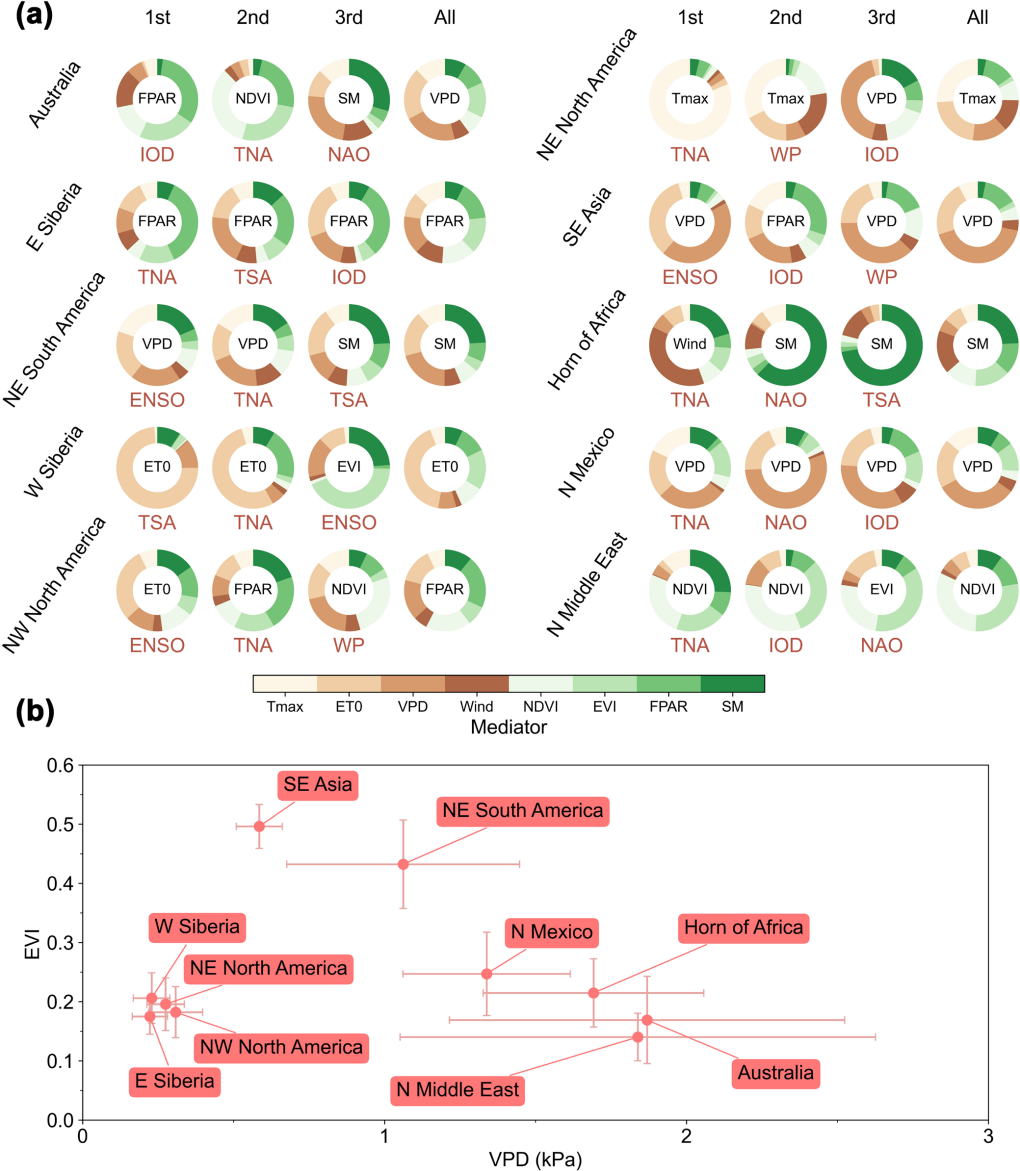
Mediators between teleconnection climate modes (TCMs) and burned area (BA). (a) Fractions of pathway contributions of a TCM on BA through mediators. The fractions for each hot spot and TCM were calculated by dividing the sum pathway contribution through each mediator by the total pathway contribution through all mediators. The mediator with the highest contribution fraction was labeled in the center. In each hot spot, the fractions from the first dominant (1st), second dominant (2nd), and third dominant (3rd) TCM, as well as all TCMs (All), are shown according to Figure 2c. (b) The regional mean vapor pressure deficit (VPD) and enhanced vegetation index (EVI) of the hot spot, indicating weather and fuel conditions, respectively. The nodes show the average values, and the error bars show the standard deviations.

The most important finding in this study is the dominant mediating effects of fuels in dryland hot spots, that is, Australia, the Horn of Africa, and the northern Middle East, as indicated by high VPD and sparse vegetation (Figure 5). In Australia, although the overall mediating effects of weather were generally stronger than those of fuels based on the summed contributions of each group (the sum of all green or brown components in Figure 5a) when all 11 TCMs were considered, two of the three most important TCMs (i.e., IOD and TNA) exhibited dominant mediating effects of fuels, particularly TNA. In the Horn of Africa, the mediating effects of fuels overall exceeded those of weather. Two of the three most important TCMs (i.e., NAO and TSA) were primarily associated with fuels mediating pathways, while only TNA showed weather‐dominant mediating effects. In the northern Middle East, the dominant mediating effects of fuels were even more pronounced. All three of the most important TCMs (i.e., TNA, IOD, and NAO) propagated their influence to BA primarily through fuel‐related pathways. In these dryland hot spots, the time lags between BA and fuel mediators are shorter than those of weather mediators (Figure S11). This suggests that TCMs may first influence weather conditions, which in turn affect fuels, thereby transmitting the impact of TCMs to BA. Nevertheless, fuels appear to serve as more effective mediators between TCMs and BA.

In northeastern South America and Southeast Asia, which are characterized by dense vegetation as indicated by higher EVI values, the overall mediating effects of weather exceeded those of fuels, particularly in Southeast Asia. In northeastern South America, the dominant mediating effects of weather were especially evident for the top two most important TCMs (i.e., ENSO and TNA). In Southeast Asia, all three of the most important TCMs (i.e., ENSO, IOD, and WP) exhibited dominant mediating effects of weather, with ENSO showing the strongest evidence. In these weather‐mediated hot spots, the time lags between weather mediators and BA were generally shorter than those in fuel‐mediated hot spots, indicating a more immediate influence of weather (Figure S11). However, in some cases, weather exhibited longer time lags than fuels (e.g., the lower quartile of weather was higher than that of fuels in northeastern South America), suggesting that the influence of weather on BA may still be transmitted through fuels, even when fuels are not the primary mediators.

In northeastern North America and northern Mexico, the mediating effects of weather outweighed those of fuels, particularly for TNA and WP in northeastern North America and for NAO in northern Mexico. In other regions, although the mediating effects of fuels did not exhibit strong overall dominance, fuels still acted as the primary mediators through which certain TCMs influenced BA. For instance, in eastern Siberia, the influence of TNA on BA was primarily transmitted via fuel‐related mechanisms. In western Siberia, the dominant mediating effects of fuels were evident. In northwestern North America, the overall mediating effects of fuels marginally exceeded those of weather. Notably, TNA, which was the second most important TCM in this region, transmitted its influence on BA predominantly through fuels.

To avoid intercorrelations within each group, we also compared the maximum contribution from each group. A consistent pattern was observed; see the green or brown component with the highest proportion in Figure 5a. In Australia, although the mediating effects of VPD were dominant overall, all three of the most important TCMs exhibited fuel‐dominant pathways (FPAR for IOD, NDVI for TNA, and SM for NAO). In the Horn of Africa, SM showed dominant overall mediating effects. Two of the most important TCMs also showed SM dominance (particularly TSA), except for TNA, which was wind‐dominant. The dominance of fuels was even more pronounced in the northern Middle East, where NDVI or EVI were the main mediating pathways. In the densely vegetated regions like northeastern South America and Southeast Asia, weather‐mediated effects dominated those of fuels. In northeastern South America, while TSA exhibited SM‐dominant pathways, ENSO and TNA were primarily mediated through VPD. In Southeast Asia, ENSO and the WP primarily influenced wildfires through VPD, whereas IOD exerted its effects mainly via FPAR. In northern Mexico, the mediating effects of fuels were weaker than those of weather, particularly VPD. In eastern Siberia, FPAR emerged as the dominant mediator for all three top TCMs (i.e., TNA, TSA, and IOD). In western Siberia, the influence of ENSO on BA was primarily mediated through EVI. In northwestern North America, FPAR mediated the influence of TNA on BA.

In addition to hot spot‐level analysis, we also examined the dominance of mediators at the grid cell scale, comparing both group sum contribution (Figure S12) and group maximum contribution (Figure S13). Both results revealed consistent patterns, with weather mediators dominating in densely vegetated hot spots and fuel mediators dominating in dryland hot spots, highlighting the robustness of this pattern. We also examined the mediators between TCMs and FRP (Figure S14). In dryland hot spots such as the northern Middle East, the Horn of Africa, and South Africa, fuel‐related mediating effects generally exceeded those of weather, with the Horn of Africa and South Africa showing overall dominance of fuel‐mediated pathways. In Southeast Asia, while the overall mediating effect of Tmax was dominant, TNA and ENSO exhibited mediation pathways primarily driven by fuels. In eastern and western Siberia, as well as northwestern North America, fuels continued to serve as key mediators between TCMs and FRP, for example, SM for NAO in eastern Siberia, and NDVI and EVI for TNA in western Siberia and northwestern North America.

## 4 Discussion

This study addressed three main questions: (a) where are the hot spots in which wildfires are highly predictable by TCMs? (b) how do TCMs influence global and regional wildfires? (c) what are the dominant mediators between TCMs and wildfires, weather or fuels? The wildfire behavior metrics used were BA and FRP. Considering the patterns obtained from BA‐based and FRP‐based analyses are largely consistent, here we only focus on the BA‐based analysis. We determined 10 hot spots, including northwestern North America, northeastern North America, northern Mexico, northeastern South America, the Horn of Africa, northern Middle East, western Siberia, eastern Siberia, Southeast Asia, and Australia. Among the 11 TCMs analyzed, TNA, TSA, ENSO, and IOD, which represent the tropical Atlantic, tropical Pacific, and tropical Indian Ocean, were identified as the most influential ones. These TCMs modulate global and regional BA through their interactions and associated impacts on atmospheric and oceanic circulations. We found that fuel mediators played a dominant role over weather mediators in dryland hot spots such as Australia, the Horn of Africa, and the northern Middle East. In contrast, in more densely vegetated hot spots like northeastern South America and Southeast Asia, weather mediators generally exerted stronger mediating effects than fuel mediators. In hot spots such as western and eastern Siberia and northwestern North America, fuel mediators could still outweigh weather mediators in modulating the influence of certain TCMs on wildfires.

### 4.1 Climate Teleconnection–Wildfire Mechanisms

The dominance of tropical climate teleconnections in modulating global BA is corroborated by studies showing that the tropical Atlantic, tropical Pacific, and tropical Indian Ocean interact with each other primarily through the Walker and Hadley circulations (Liu et al. 2023; Meehl et al. 2020), with their interactions further modulated by the NAO (Ding et al. 2023; Yang et al. 2021). The ENSO dominance in western Siberia and northwestern North America can be explained by the fact that the tropical Pacific impacts high latitudes via the interactions with the tropical Indian Ocean, the tropical Atlantic, and the North Pacific (Hu and Fedorov 2019; Liao and Wang 2021). The dominance of ENSO in northeastern South America and Southeast Asia has been well studied (Cardil et al. 2023; Chen, Morton, et al. 2016). In these regions, the time lags between ENSO and BA are typically short (0–8 months), with VPD playing a dominant mediating role (Figures 4 and 5). This suggests that wildfires respond relatively quickly to ENSO events primarily through weather‐related pathways. In these regions where fuels are abundant while fire‐conducive weather is limited, decreased precipitation and increased evapotranspiration in El Niño years increase the likelihood of large fires, which is evident by the positive correlations between ENSO and BA. Different from northeastern South America and Southeast Asia, the correlation between ENSO and BA in Australia is negative (Figure S8). The long time lags and negative correlations suggest that ENSO influences fuel accumulation, as reflected by the dominant mediating role of EVI. During El Niño events, reduced precipitation limits SM and hence fuels in Australian savannas, where wildfires are primarily fuel‐limited (Le and Bae 2022; Qu et al. 2023).

The dominance of IOD is shown in Australia, western Siberia, northeastern North America, Southeast Asia, northern Mexico, and the northern Middle East. The positive phase of IOD results in reduced precipitation and increased evapotranspiration in these regions, thereby increasing wildfire risk, which is supported by the positive correlations and short time lags between IOD and BA. The influence of IOD on BA in the northern mid‐ to high latitudes can be attributed to two distinct teleconnection pathways, which resemble those of ENSO, as IOD and ENSO have a robust causal relationship (Hardiman et al. 2020; Le and Bae 2019). The first is a tropospheric pathway, in which anomalies originating from the Indian Ocean propagate to the North Atlantic via a Rossby wave train that passes over the Pacific and Atlantic (Hardiman et al. 2020). The second is a stratospheric pathway, whereby the Rossby wave train from the Indian Ocean weakens the Aleutian cyclone over the North Pacific, altering planetary wave patterns and strengthening the stratospheric polar vortex, ultimately favoring the development of a positive NAO (Hardiman et al. 2020). In the northern Middle East, the IOD is positively correlated with BA, with short time lags (approximately 0 and 7 months), likely reflecting its influence on fuel buildup, as indicated by the dominant mediating role of NDVI. In northeastern South America, the positive correlation between IOD and BA is mainly mediated by SM (Figures S8 and S10), likely due to the connection between IOD and ENSO and the impacts of ENSO on regional SM (Le and Bae 2019, 2022).

Some TCMs, like TNA, TSA, and NAO, are pivotal in propagating signals from tropical ocean anomalies across vast distances and timescales. The tropical Atlantic dominance is evident in all hot spots except for Southeast Asia. El Niño events heat both the tropical Indian Ocean and tropical Atlantic, with the warming pattern persisting longer over the tropical Atlantic (Matsumura and Kosaka 2019). The Rossby wave from the tropical Atlantic reaches Eurasia through the North Atlantic, thereby warming the high latitudes of the Northern Hemisphere (Fuentes‐Franco et al. 2022). The tropical Atlantic also exerts a strong influence on wildfires in the Horn of Africa, the northern Middle East, northern Mexico, and northeastern South America. The warm and cold SST anomalies over the tropical Atlantic force the north–south shift of the Intertropical Convergence Zone (ITCZ), resulting in precipitation anomalies in these regions surrounding the tropical Atlantic (Chen et al. 2011). The propagating effect of TNA and TSA, along with their influence on ITCZ, may explain the dominance of TNA and TSA in global BA, particularly in western and eastern Siberia (Figure S3). This global dominance of tropical Atlantic TCMs is also supported by a previous study (Cardil et al. 2023).

The importance of NAO in modulating global wildfires is consistent with the expectation that the NAO can modulate the coupling between the tropical oceans by altering the path and strength of midlatitude atmospheric circulations, such as the jet stream and Rossby waves (Ding et al. 2023; Yang et al. 2021) as well as oceanic circulation (Bellucci et al. 2008). When the NAO is in its positive phase, the strengthening of winds over the North Atlantic alters the Walker circulation, which in turn regulates the interactions between the tropical ocean systems (Ding et al. 2023). These cross‐regional couplings may explain the influence of NAO over hot spots that are adjacent to tropical oceans, such as Australia, the Horn of Africa, and northern Mexico. In the northern Middle East, NAO and BA are negatively correlated in a dominant time lag of around 9 months (Figure 4). This aligns with the expectation that the negative phase of NAO lowers the pressure gradient between Icelandic Low and Azores High, resulting in weaker westerlies, thus conducive to the transport of vapor to the areas surrounding the Mediterranean (Wang et al. 2014) and therefore fuel accumulation, which is evident by the dominant mediating effects of EVI. NAO also plays an important role in eastern Siberia, ranked as the fourth most important TCM there (Figure 2c). The positive phase of NAO in winter favors the vapor input from the North Pacific in spring, while the accompanying Arctic sea ice loss prevents vapor transport from Siberia to the Laptev Sea and thus decreases summer precipitation (Zhu et al. 2021). This may explain the negative correlations in eastern Siberia between NAO and BA around the time lag of 19 months and positive correlations around the time lag of 23 months (Figure S8). The longer time lags (more than 1 year) in eastern Siberia agree with the expectation of long moisture memory and carry‐over effect (the growth of vegetation is influenced by its previous states) of forests (Lian et al. 2021).

### 4.2 Fuels Mediating Climate Teleconnection–Wildfire

Fuel buildup and continuity are related to weather conditions and land cover changes like cropland expansion in sub‐Saharan Africa (Andela and van der Werf 2014) and flammable species invasion in eastern Siberia (McCarty et al. 2020). This suggests that in regions where the mediating effects of fuels are dominant, weather mediators continue to act as indirect controls, with their effects transmitted by fuels. In other words, weather mediators influence wildfires through both immediate and delayed effects. The immediate effects reflect direct wildfire responses to weather variability driven by TCMs, while the delayed effects represent lagged wildfire responses.

In more densely vegetated hot spots such as northeastern South America and Southeast Asia, weather mediators generally exerted stronger mediating effects on wildfires than fuel mediators (Figure 5). In these hot spots, the time lags between weather mediators and wildfires were typically shorter than those of fuel mediators, indicating more immediate effects of weather mediators (Figure S11). However, in some cases, the time lags for weather mediators exceeded those of fuel mediators, suggesting more delayed effects; in other words, the influence of weather may have been indirectly transmitted through fuel mediators. Nonetheless, fuel mediators alone are insufficient to mediate the influence of TCMs on wildfires in these regions and thus do not serve as the primary mediating pathway.

The delayed influence of weather mediators on wildfires is evident in dryland hot spots such as Australia, the Horn of Africa, and the northern Middle East, where fuel‐related mediators play a key role in mediating the influence of TCMs on wildfires (Figure 5). By analyzing time lags, we found that in these regions, fuel variability and its delayed effects on wildfires were largely driven by weather variability that was induced by TCMs. This is supported by the finding that time lags associated with fuel mediators were generally shorter than those associated with weather variables (Figure S11). This does not imply that the dominant mediating effects of fuels are spurious. Rather, this suggests that fuel mediators serve as effective proxies for vegetation dryness or biomass, capturing the lagged effects of weather mediators on wildfires. In addition to dryland hot spots, fuel mediators also played a dominant mediating role for all three most important TCMs in eastern Siberia. In other hot spots like western Siberia and northwestern North America, fuel mediators could still act as a primary pathway through which certain TCMs modulate wildfires.

Previous studies that investigated the influences of TCMs on wildfires regionally or globally did not provide a comprehensive analysis of the underlying pathways, or only focused on the immediate effects of weather mediators. The omission of fuel mediators may lead to either an overestimation or underestimation of the influence of TCMs. In eastern Siberia, for example, FPAR is the primary mediator linking TNA, TSA, and IOD with BA. Quantifying the influence of TCMs on BA through widely used weather mediators (e.g., Tmax and VPD) may incorrectly attribute the effect of anthropogenic warming to that of TCMs and thus overestimate it. This case of overestimation may be solved by considering fuel mediators within the wildfire attribution framework. In the drylands of Australia, after a 3‐year La Niña (2020–23), the fuel load was increased due to the increased precipitation (AFAC 2023). Following the onset of El Niño in 2023, drought conditions emerged and dried up fuels. This combination of increased fuel load and dryness may have potentially increased the risk of extreme wildfires (Zhang 2023). However, if only El Niño‐induced droughts or heatwaves are considered, studies may underestimate the fire risk as the increased fuels stemming from the preceding La Niña are overlooked. Such underestimation can be mitigated by accurately identifying the time lags, the sign of the relationships (positive or negative), and the mediators between ENSO and BA.

### 4.3 Implications, Limitations, and Future Directions

Anthropogenic warming may increase the magnitude of ENSO, and therefore TNA and PNA, due to their interactions (Cai et al. 2023, 2022). The strengthened El Niño event may be followed by a multi‐year La Niña because of the large heat budget (Cai et al. 2023; Geng et al. 2023). The transition from a 3‐year La Niña (2020–2022) to a strong El Niño (2023) combined with anthropogenic warming made 2023 the warmest year on record (Raghuraman et al. 2024). The combined effects of anthropogenic warming and intensified climate teleconnection patterns may exacerbate wildfire conditions, posing increasing challenges to current fuel management strategies and wildfire mitigation policies. We proposed a new climate teleconnection–wildfire framework that systematically integrates the spatial, temporal, and mechanistic aspects of their relationship. By addressing these three aspects, the framework offers a more comprehensive understanding of how large‐scale TCMs influence wildfire behaviors across different regions and timescales.

Ten hot spots where TCMs show higher‐than‐average BA predictability were determined, including northwestern North America, northeastern North America, northern Mexico, northeastern South America, the Horn of Africa, northern Middle East, western Siberia, eastern Siberia, Southeast Asia, and Australia. Several cold spots where TCMs show lower‐than‐average BA predictability were also determined, including the Central United States, the southern equatorial region of sub‐Saharan Africa, northern and southeastern China, and northwestern Indochina. These hot (cold) spots indicate regions where wildfires are (are not) modulated by TCMs, suggesting that attribution and prediction based on TCMs may be effective (ineffective) in these areas.

As successive strong climate teleconnection events are projected to become more frequent in the future (Geng et al. 2023), accurately accounting for time lags becomes increasingly important. Failing to distinguish the influence between successive climate teleconnection events can conflate their individual impacts and compromise the reliability of wildfire predictions. Our framework can effectively disentangle these successive or overlapping effects by identifying time lags, a critical step toward improving the accuracy of wildfire attribution and prediction.

Accurately identifying teleconnection mechanisms enables early warning systems that can inform governments and the public, allowing interventions to mitigate the impacts of extreme wildfires. Through a pathway analysis, we quantified, for the first time, the mediating effects of weather and fuels, highlighting the critical role of fuel mediators in transmitting the influence of TCMs to wildfires, especially in dryland hot spots like Australia, the Horn of Africa, and the northern Middle East. Our findings underscore that neglecting the mediating effects of fuels can result in significant overestimation or underestimation of the impacts of TCMs on wildfires. This stresses the need to refine fuel management strategies and integrate fuel conditions into teleconnection‐related wildfire attribution and prediction efforts.

In the future, the following investigations could further narrow the uncertainties and limitations of this study. First, we classified mediators into weather‐ and fuel‐related, but ignitions from human behavior and lightning are also important for wildfires (Janssen et al. 2023; Mariani et al. 2018) and may not be represented adequately by the mediators used. Second, the location and timing of a TCM event are important. In certain circumstances, the central position of SST anomalies is more related to wildfires than their intensity. For example, wildfires in Indonesia tend to be larger during Eastern Pacific El Niño and smaller during Central Pacific El Niño (Chen, Lin, et al. 2016). Third, a longer BA time series would be beneficial in minimizing the impact of extreme TCM events. Last, as anthropogenic warming continues, the impacts of TCMs may be amplified and reach potential tipping points (Duque‐Villegas et al. 2019), which should be considered.

## 5 Conclusion

This study determined 10 hot spots where BA was highly predictable by TCMs. Hot spots with the highest probability were Australia and eastern Siberia. Globally, BA in approximately 25.4% of burnable regions was predictable by a single TCM. Climate teleconnections originating from the tropical Atlantic, tropical Pacific, and tropical Indian Ocean played the most significant roles in modulating global BA. These tropical oceans interact with each other and influence wildfires globally via atmospheric and oceanic circulations. TNA and TSA propagate anomalies from the tropical Atlantic to the high latitudes of the Northern Hemisphere, particularly affecting western and eastern Siberia. Combined with their influence on the north–south shifts of ITCZ, they emerged as the most influential TCMs. In dryland hot spots like Australia, the Horn of Africa, and the northern Middle East, the mediating effects of fuels dominate those of weather. In more densely vegetated hot spots like northeastern South America and Southeast Asia, the mediating effects of weather generally exceeded the effects of fuels. In other hot spots, the fuel mediators could still act as a primary pathway through which certain TCMs modulate wildfires. To the best of our knowledge, this was the first attempt to comprehensively analyze the global climate teleconnection–wildfire pathways, considering both weather and fuel mediators. Our study highlights the important role fuel mediators play in connecting TCMs and BA, especially in dryland hot spots. It provides new insights into developing refined fuel management strategies and wildfire attribution and prediction frameworks, which are crucial in the context of climate change and the resulting changes in teleconnection patterns.

## Author Contributions

**Yuquan Qu:** conceptualization, formal analysis, methodology, writing – original draft. **Sander Veraverbeke:** formal analysis, writing – review and editing. **Diego G. Miralles:** formal analysis, methodology, writing – review and editing. **Jingfang Fan:** formal analysis, writing – review and editing. **Harry Vereecken:** conceptualization, methodology, supervision, writing – review and editing. **Carsten Montzka:** conceptualization, methodology, supervision, writing – review and editing.

## Conflicts of Interest

The authors declare no conflicts of interest.

## Supporting information


**Data S1:** gcb70406‐sup‐0001‐Supinfo.pdf.

## Data Availability

The data and code that support the findings of this study are openly available in Zenodo at https://doi.org/10.5281/zenodo.16277102. MODIS data were obtained from the NASA Land Processes Distributed Active Archive Center at https://doi.org/10.5067/MODIS/MCD64A1.061, https://doi.org/10.5067/MODIS/MYD14A1.061, https://doi.org/10.5067/MODIS/MOD13C2.061, and https://doi.org/10.5067/MODIS/MCD15A3H.061. ERA5 data were obtained from the Copernicus Climate Change Service Climate Data Store at https://doi.org/10.24381/cds.f17050d7. The AO, AAO, NAO, TNA, TSA, EAWR, PNA, ENSO, and WP indices were obtained from the National Oceanic and Atmospheric Administration (NOAA) at https://psl.noaa.gov/data/climateindices/list/, and the IOD index at https://psl.noaa.gov/data/timeseries/month/. The POL index was obtained from the Koninklijk Nederlands Meteorologisch Instituut Climate Explorer at https://climexp.knmi.nl/selectindex.cgi (Polar/Eurasia patterns).
