# Supplementary material for: Fuels Mediate the Influence of Climate Teleconnections on Wildfires in Dryland Ecosystems
Source: Glob Chang Biol. 2025 Aug 6;31(8):e70406. doi: 10.1111/gcb.70406 (PMC12326949; doi:10.1111/gcb.70406)
Supplement: Supplementary file 1 — Data S1: gcb70406‐sup‐0001‐Supinfo.pdf. [file GCB-31-e70406-s001.pdf]

Supplementary Information for

Fuels mediate the influence of climate teleconnections on wildfires in dry ecosystems

Yuquan Qu[1, 2*], Sander Veraverbeke[2, 3], Diego G. Miralles[4], Jingfang Fan[5, 6], Harry Vereecken[1], Carsten Montzka[1]

1. Institute of Bio- and Geosciences: Agrosphere (IBG-3), Forschungszentrum Jülich GmbH, Jülich, Germany.
2. Faculty of Science, Vrije Universiteit Amsterdam, Amsterdam, Netherlands.
3. School of Environmental Sciences, University of East Anglia, Norwich, United Kingdom.
4. Hydro-Climate Extremes Lab, Ghent University, Ghent, Belgium.
5. School of Systems Science, Beijing Normal University, Beijing, China.
6. Potsdam Institute for Climate Impact Research, Potsdam, Germany.

1 Data

1.1 Fire radiative power

Fire radiative power (FRP) data used to characterize fire intensity was obtained from MYD14A1 (version 6.1) (Giglio, 2021) at 1 km spatial resolution and daily temporal resolution. We excluded FRP values with large Moderate Resolution Imaging Spectroradiometer (MODIS) scan angles (θ>50°) due to potential uncertainty and normalized the raw FRP values for pixel size. The equations are shown below. Since FRP follows a power-law distribution (Wooster & Zhang, 2004), summary statistics such as the median and percentiles are more appropriate than the mean. In this study, the daily 1 km normalized FRP was resampled to a monthly 1° resolution based on its 95th percentile.

$$FRP_{normalized} = \frac{FRP}{d_S \times d_T} \qquad \text{Equation. 1}$$

$$d_S = R_e \times s \times (\frac{\cos\theta}{\sqrt{(\frac{R_e}{r})^2 - (\sin\theta)^2}} - 1) \qquad \text{Equation. 2}$$

$$d_T = r \times s \times \left(\cos\theta - \sqrt{\left(\frac{R_e}{r}\right)^2 - (\sin\theta)^2}\right) \qquad \text{Equation. 3}$$

$$r = R_e + h \qquad \text{Equation. 4}$$

$$\theta = s \times (sample - 676.5) \qquad \text{Equation. 5}$$

where FRP is the raw FRP value. $FRP_{normalized}$ is the FRP normalized by pixel size, in MW/km^2. R_e is the Earth's radius, which is 6378.137 km. s is 0.0014184397. h is the satellite altitude, which is 705 km. θ is the scan angle. $sample$ is the position of the fire pixel within the scan.

1.2 Vapor pressure deficit

Vapor pressure deficit (VPD) indicates the ability of the atmosphere to extract water from the environment by measuring the difference between the amount of water vapor in the air and how much water vapor it can hold when the air is saturated. In this study, VPD was calculated using minimum and maximum 2m air temperature and dewpoint temperature from ERA5. The equations are shown below.

$$\mathrm{VPD} = \mathrm{e_s} - \mathrm{e_a} \qquad \text{Equation. 6}$$

$$e_s = \frac{(e_{tmax} + e_{tmin})}{2} \quad \text{Equation. 7}$$

$$e_a = 0.6108 \times \exp\left(\frac{17.27 \times T_{dew}}{T_{dew} + 237.3}\right) \quad \text{Equation. 8}$$

$$e_{tmax} = 0.6108 \times \exp\left(\frac{17.27 \times T_{max}}{T_{max} + 237.3}\right) \quad \text{Equation. 9}$$

$$e_{tmin} = 0.6108 \times \exp\left(\frac{17.27 \times T_{min}}{T_{min} + 237.3}\right) \quad \text{Equation. 10}$$

where e_s is saturation vapor pressure and e_a is actual vapor pressure. T_{dew} is dewpoint temperature, T_{max} is maximum air temperature, and T_{min} is minimum air temperature.

2 Methods

2.1 Selection of partial least squares regression

In this study, the regression between a teleconnection climate mode (TCM) and burned area (BA) included 25 independent variables, with a time series length of 264 months (22 years). Meanwhile, this study focused on BA predictability rather than causal inference, considering that TCMs function as a complex interacting system. Partial least squares regression was therefore applied to maximize BA predictive performance, especially in the context of high dimensionality introduced by time-lagged TCMs, where traditional regression methods may fail.

2.2 Getis-Ord Gi* statistic hot spot analysis

The Getis-Ord Gi* hot spot analysis identifies areas where local cluster statistics are significantly higher or lower than the global average, indicating hot or cold spots. The outputs of the Getis-Ord Gi* statistic include z-scores, p-values, and Gi_Bin. The z-scores represent the clustering intensity. The p-values indicate whether the difference between local clusters and global grid cells is statistically significant. They are used to determine if the null hypothesis (complete spatial stochasticity) is rejected. The Gi_Bin is a combination of z-scores and p-values, it is used to classify the local clusters into hot spots and cold spots. A Gi_Bin value of 1, 2, and 3 (-1, -2, and -3) indicates a hot (cold) spot where the local cluster is statistically higher (lower) than global grid cells with a 0.1, 0.05, and 0.01 significance level.

2.3 Justification and assumptions of the pathway analysis

As the coefficient of determination indicates the fraction of variation in the dependent that can be explained by the independent(s), it is feasible to multiply two coefficients of determination. A mediator contributes to BA through two distinct components: a TCMs-independent component and a TCMs-dependent component. We hypothesized that when the TCMs-independent component significantly contributes to BA, the TCMs-dependent component also has a notable impact, assuming the anomalies of mediator driven by TCMs are larger in magnitude. If the TCMs-dependent component cannot significantly impact BA, for example, when the impacts of TCMs on BA occur during the non-fire seasons and fail to alter fuel conditions, the predictability of BA by TCMs would be limited. However, this case had been ruled out in our analysis by checking p_1.

3 Interactions among tropical oceans

From a dynamical perspective, sea surface temperature (SST) anomalies in these tropical oceans drive atmospheric responses through the Walker and Hadley circulation (Wang, 2004). The Walker circulation involves ascending and descending air currents over the tropics, which influence the strength and direction of trade winds in the tropical troposphere. These wind anomalies, in turn, induce large-scale climate responses globally (McGregor et al., 2014). During an El Niño event in the tropical Pacific, the Walker circulation weakens, leading to SST anomalies in both the Indian Ocean and the Atlantic. These SST changes affect atmospheric circulation patterns, which can feed back to the Pacific, establishing complex bidirectional interactions between these basins (Cai et al., 2019). Thermodynamically, the SST differences between the tropical oceans directly affect the distribution of latent and sensible heat fluxes. This alters the thermodynamic state of the atmosphere and oceans and leads to variations in ocean–atmosphere heat exchanges, which are crucial in shaping the energy distribution within climate systems. The heat absorbed or released by the oceans modulates the development and maintenance of energy gradients in the atmosphere, which then influence the spatial and temporal evolution of teleconnection patterns (Rind, Chandler, Lerner, Martinson, & Yuan, 2001) and has downstream effects on global climate patterns, particularly influencing wave patterns at mid and high latitudes.

Reference

Cai, W., Wu, L., Lengaigne, M., Li, T., McGregor, S., Kug, J. S., . . . Chang, P. (2019). Pantropical climate interactions. *Science, 363*(6430). doi:10.1126/science.aav4236

Giglio, L., Justice, C. (2021). *MODIS/Aqua Thermal Anomalies/Fire Daily L3 Global 1km SIN Grid V061*.

McGregor, S., Timmermann, A., Stuecker, M. F., England, M. H., Merrifield, M., Jin, F.-F., & Chikamoto, Y. (2014). Recent Walker circulation strengthening and Pacific cooling amplified by Atlantic warming. *Nature Climate Change, 4*(10), 888-892. doi:10.1038/nclimate2330

Rind, D., Chandler, M., Lerner, J., Martinson, D. G., & Yuan, X. (2001). Climate response to basin-specific changes in latitudinal temperature gradients and implications for sea ice variability. *Journal of Geophysical Research: Atmospheres, 106*(D17), 20161-20173. doi:10.1029/2000jd900643

Wang, C. (2004). ENSO, Atlantic climate variability, and the Walker and Hadley circulations. In H. F. Diaz & R. S. Bradley (Eds.), *The Hadley Circulation: Present, Past and Future* (pp. 173-202). Dordrecht: Springer Netherlands.

Wooster, M. J., & Zhang, Y. H. (2004). Boreal forest fires burn less intensely in Russia than in North America. *Geophysical Research Letters, 31*(20). doi:10.1029/2004gl020805

4 Figures

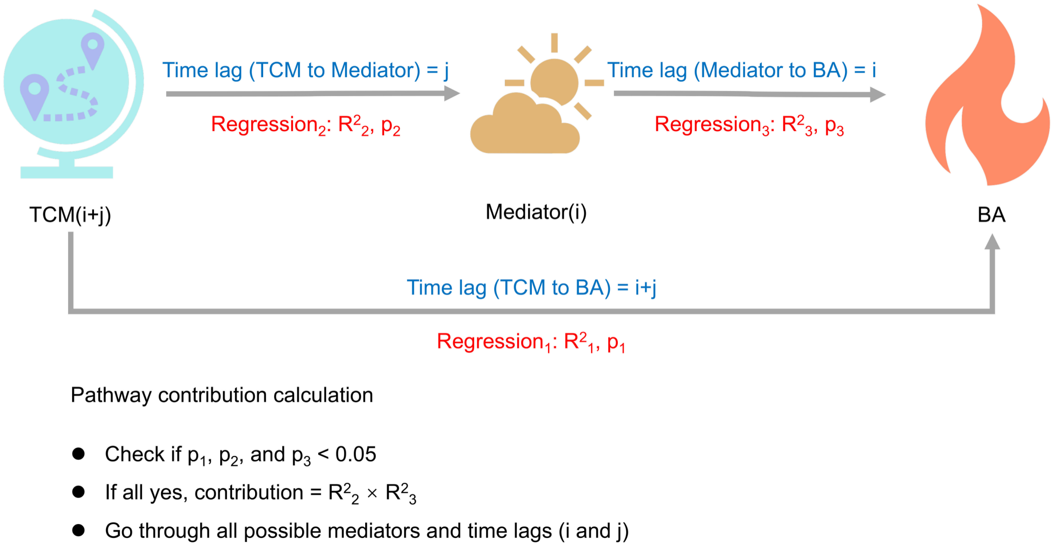

Figure S1. Flowchart of the pathway contribution calculation. TCM is the teleconnection climate mode. BA is the burned area. R^2 is the coefficient of determination between the predicted and observed dependent variable. p is from the F-statistic of the regression. i and j are the time lags. Note that i and j range from 0 to 24, and i+j should be no more than 24.

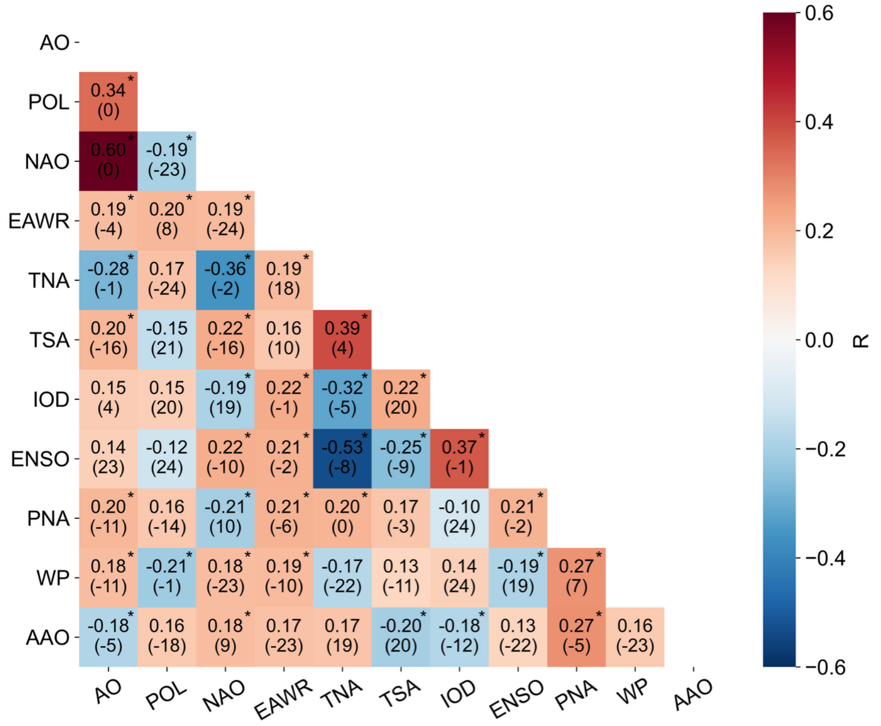

Figure S2. Correlations among teleconnection climate modes (TCMs). The heatmap displays the strongest correlations (based on absolute values) along with their corresponding time lags (in parentheses, with positive values denoting the row TCM leading the column TCM) between a pair of TCMs. The symbol "*" indicates that the correlations are significant (p<0.05).

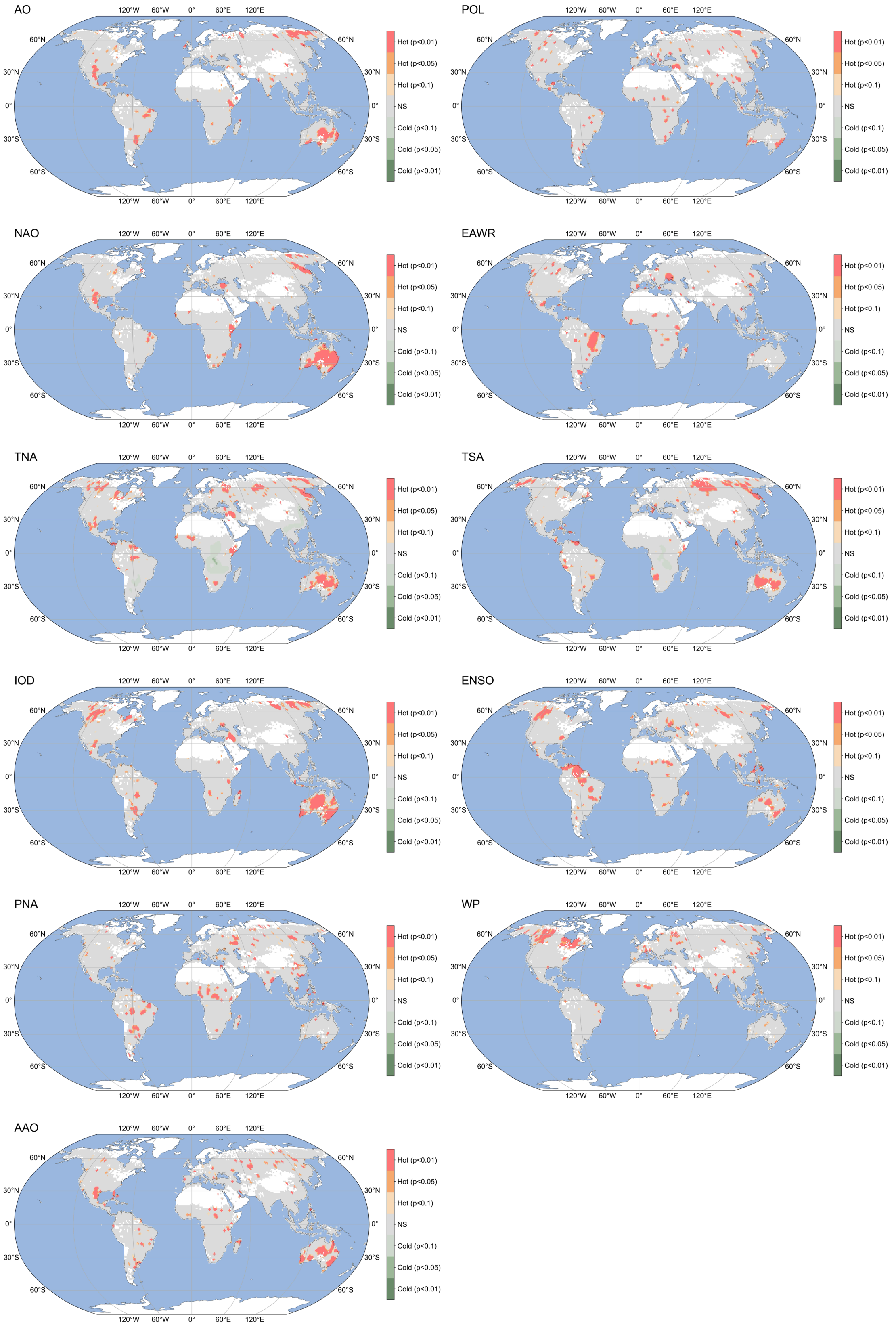

Figure S3. Hot/cold spots where the teleconnection climate mode shows high/low burned area predictability. Like Figure 1c, but only a single time-lagged teleconnection climate mode was used.

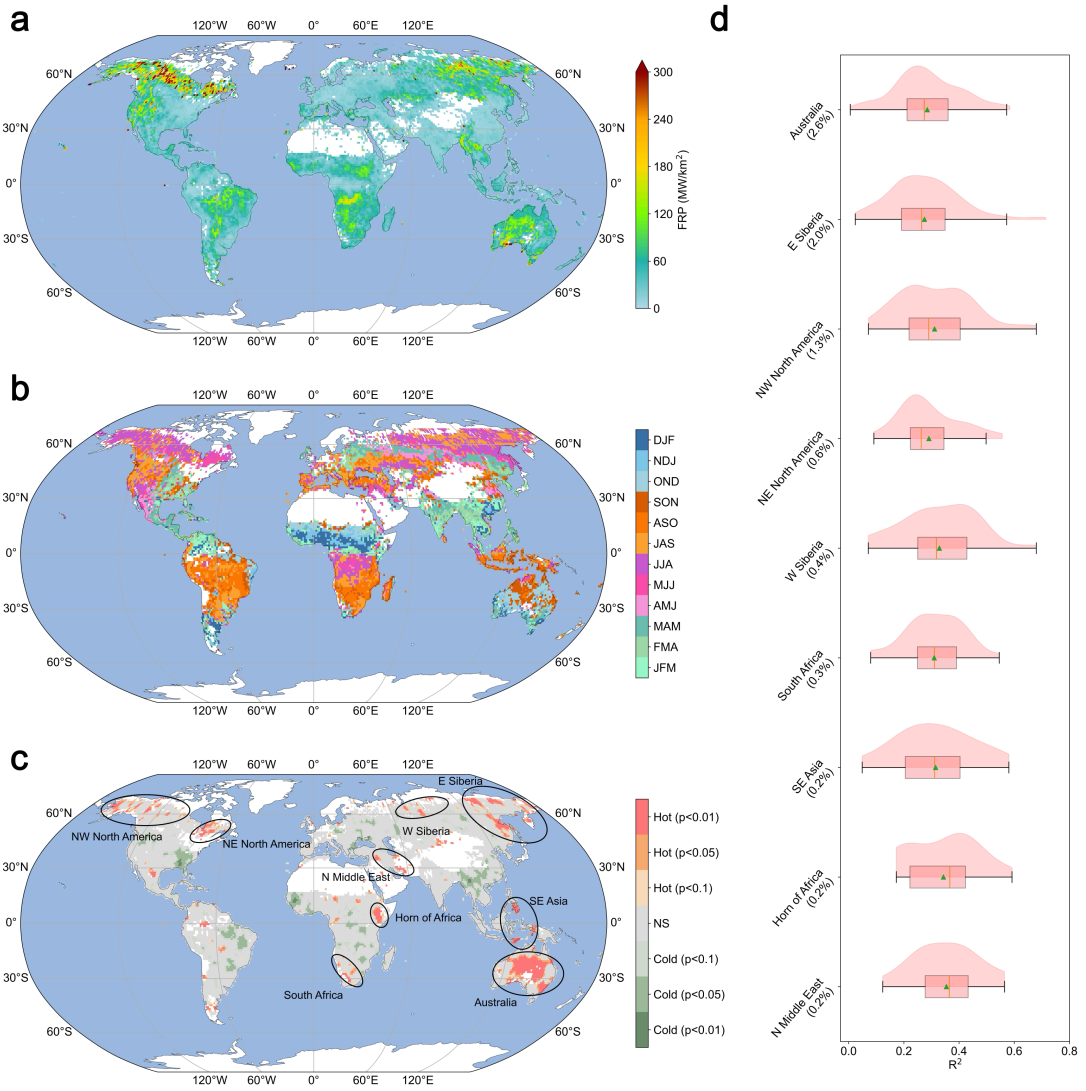

Figure S4. Global fire radiative power pattern and hot spots where fire radiative power is highly predictable by teleconnection climate modes. Like Figure 1, but for fire radiative power.

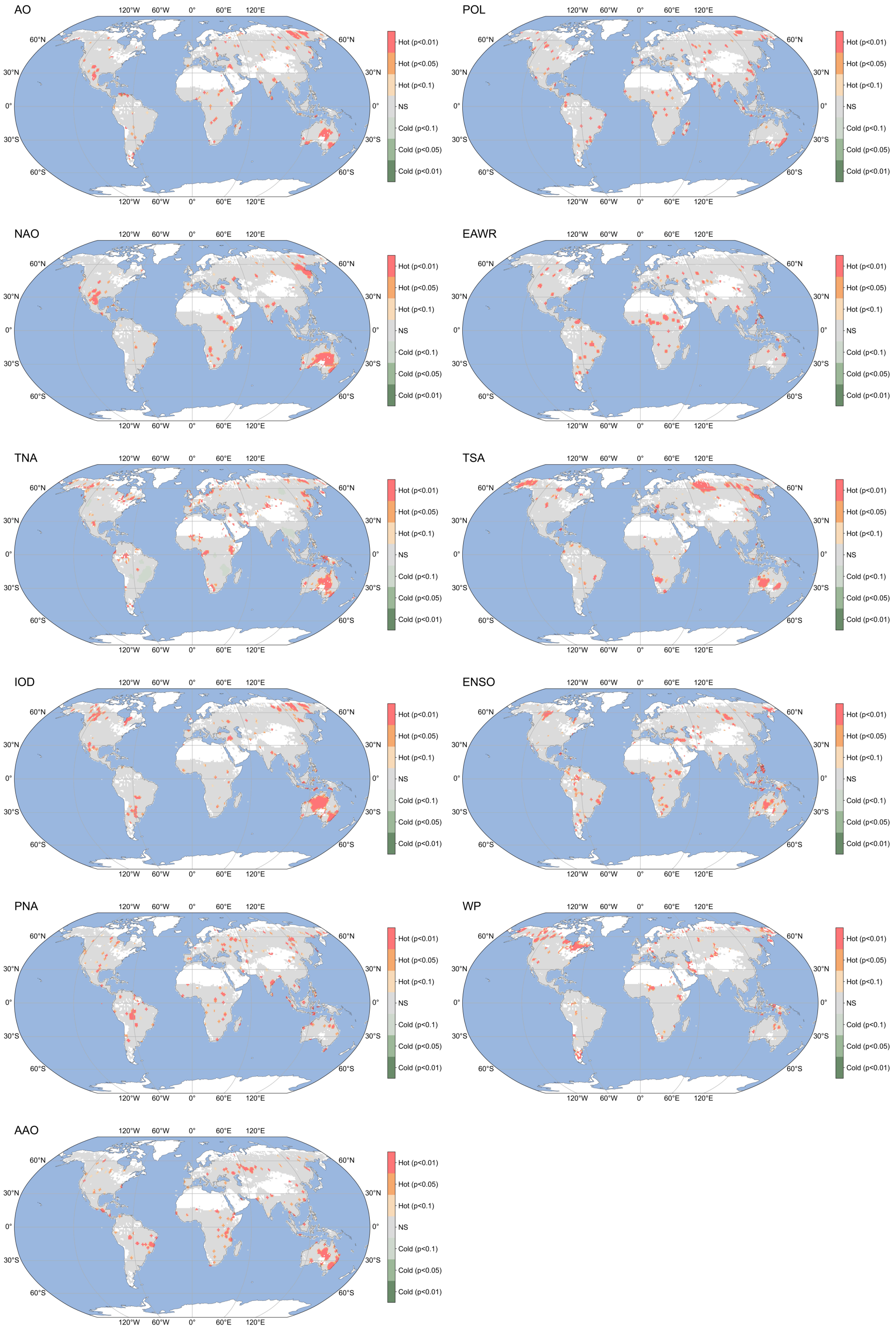

Figure S5. Hot/cold spots where the teleconnection climate mode shows high/low fire radiative power predictability. Like Figure S3, but for fire radiative power.

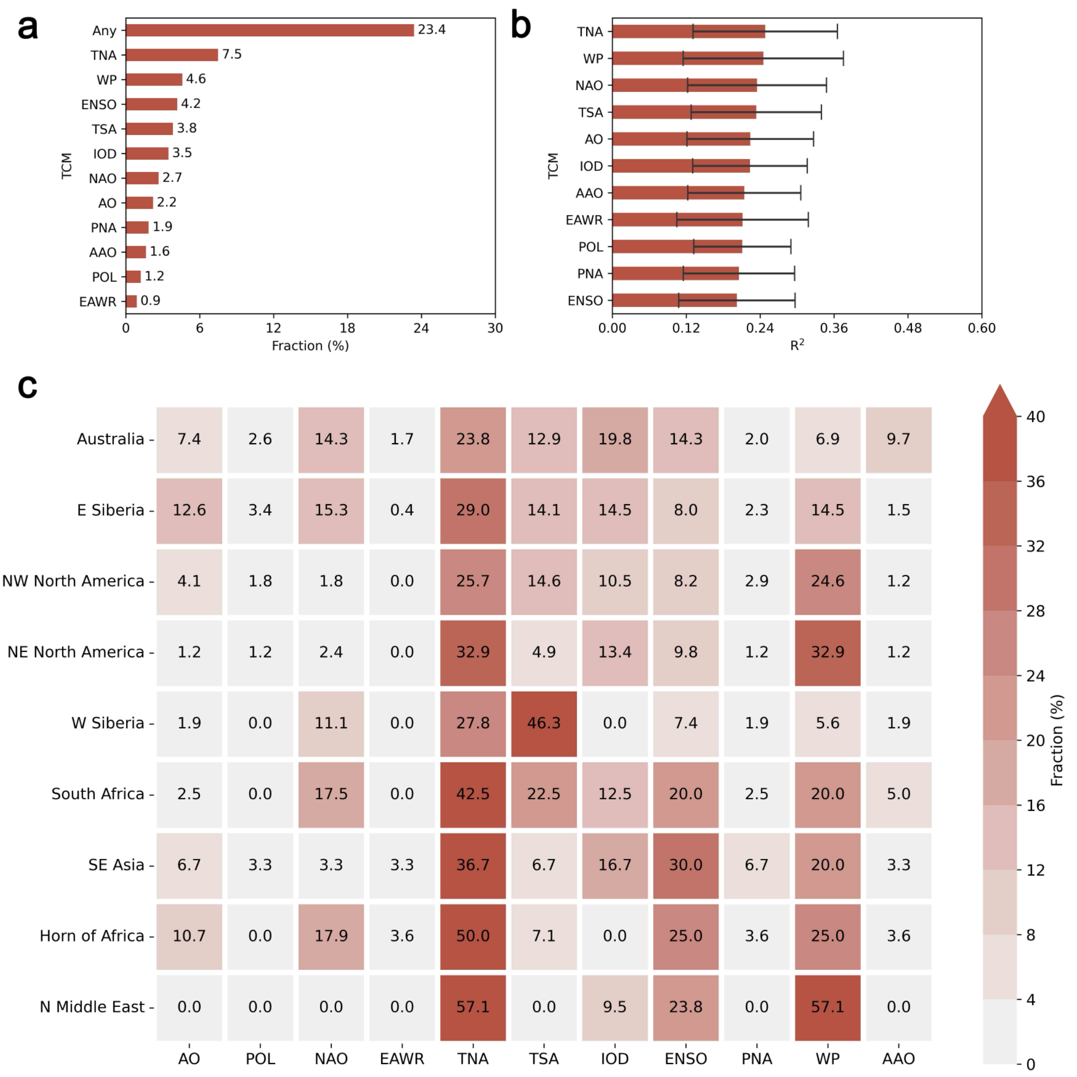

Figure S6. The importance of teleconnection climate modes in predicting fire radiative power. Like Figure 2, but for fire radiative power.

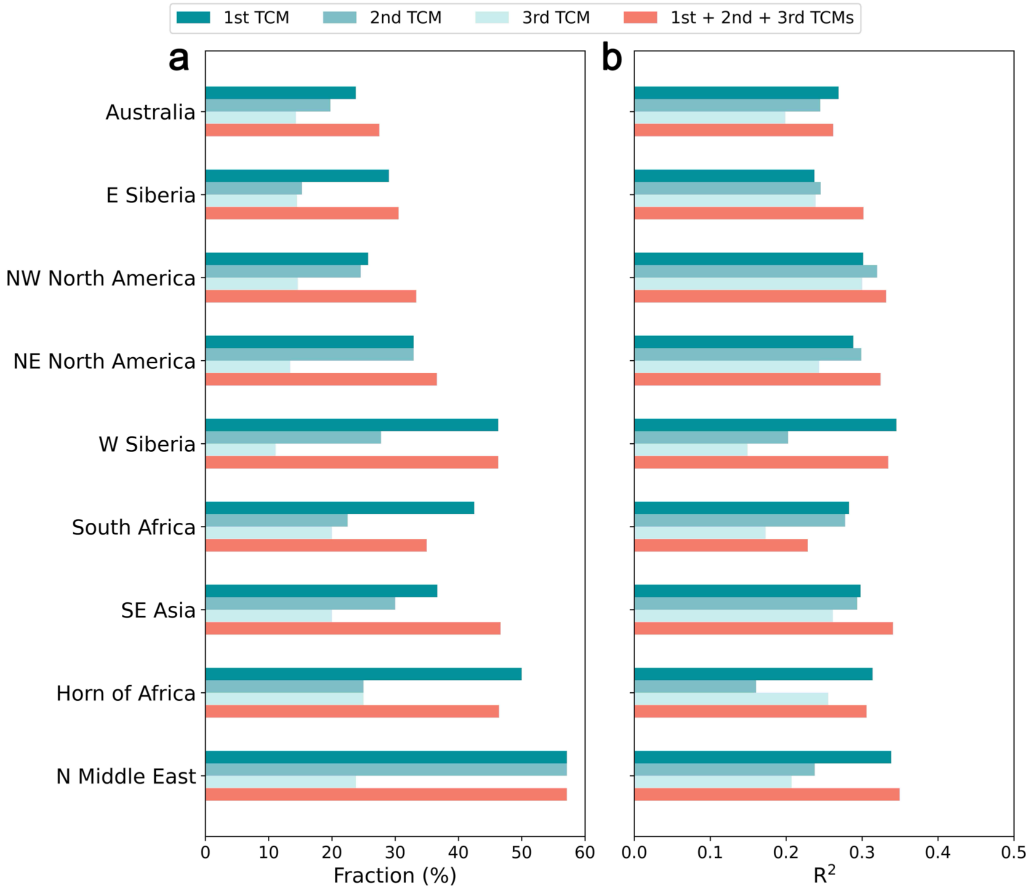

Figure S7. Fire radiative power predictability in hot spots driven by teleconnection climate modes. Like Figure 3, but for fire radiative power.

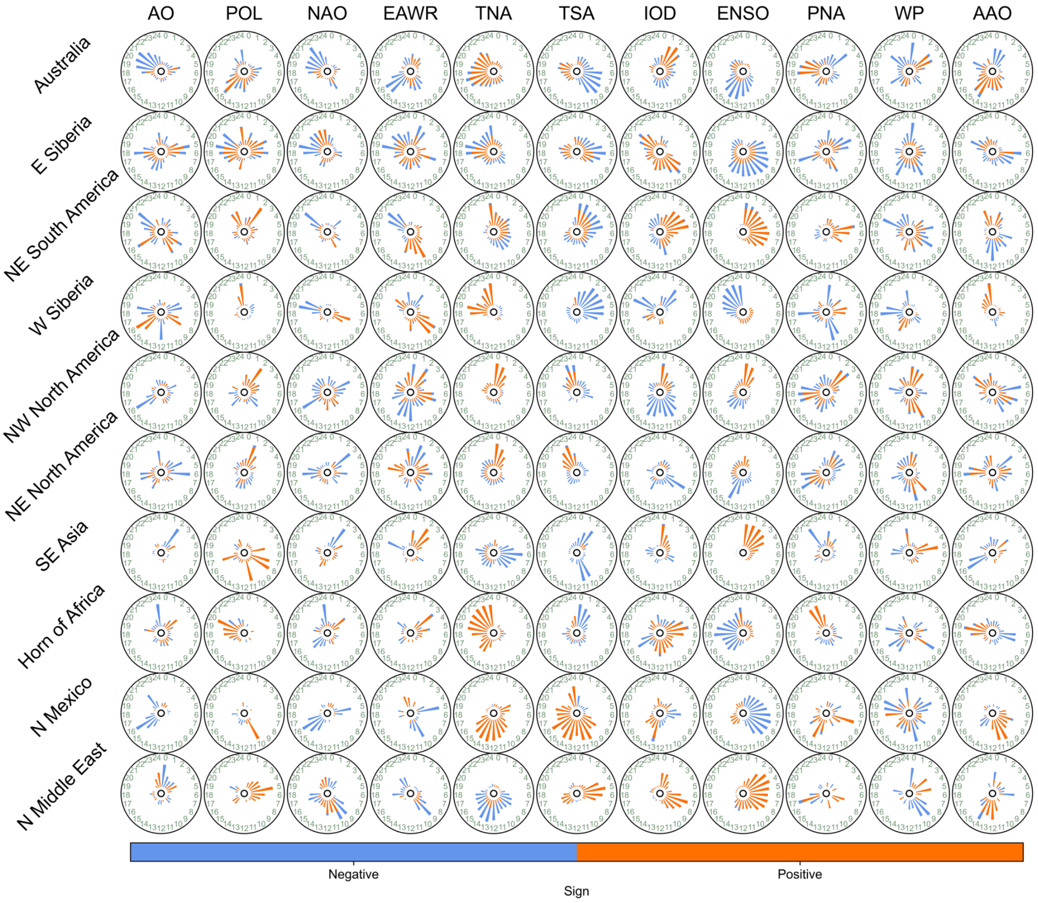

Figure S8. Time lags between teleconnection climate modes and burned area in hot spot regions.

Like Figure 4, but for all hot spot regions and teleconnection climate modes.

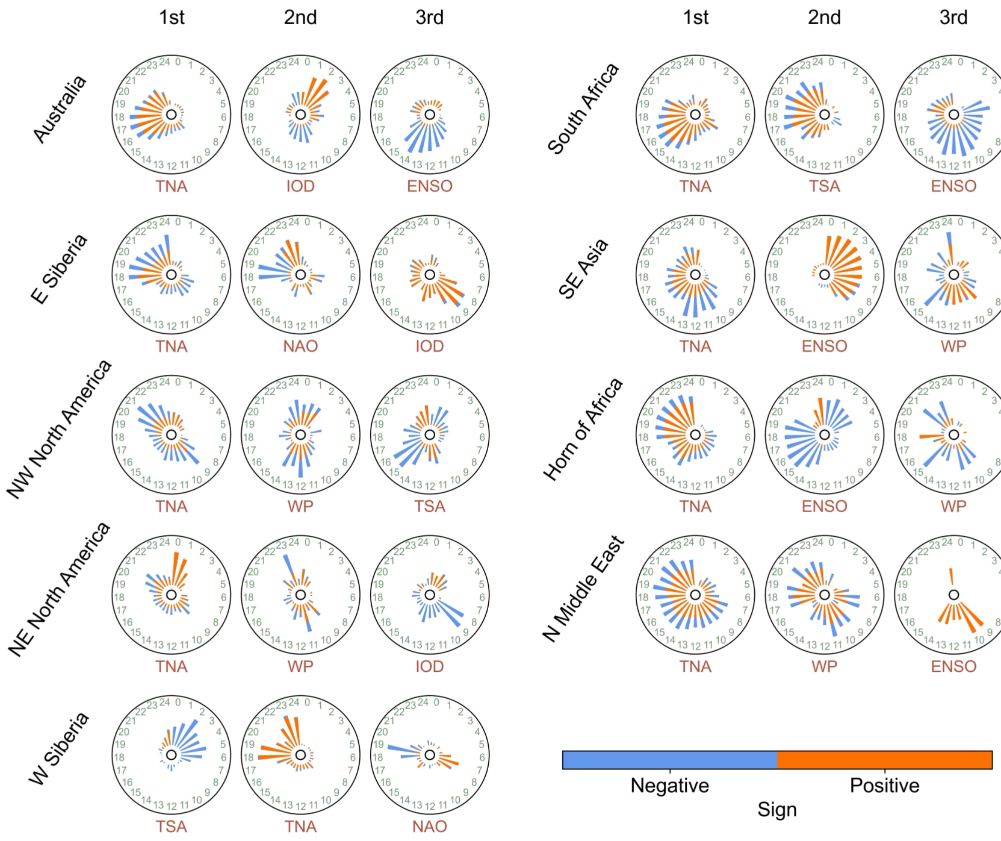

Figure S9. Time lags between teleconnection climate modes and fire radiative power in hot spots.

Like Figure 4, but for fire radiative power.

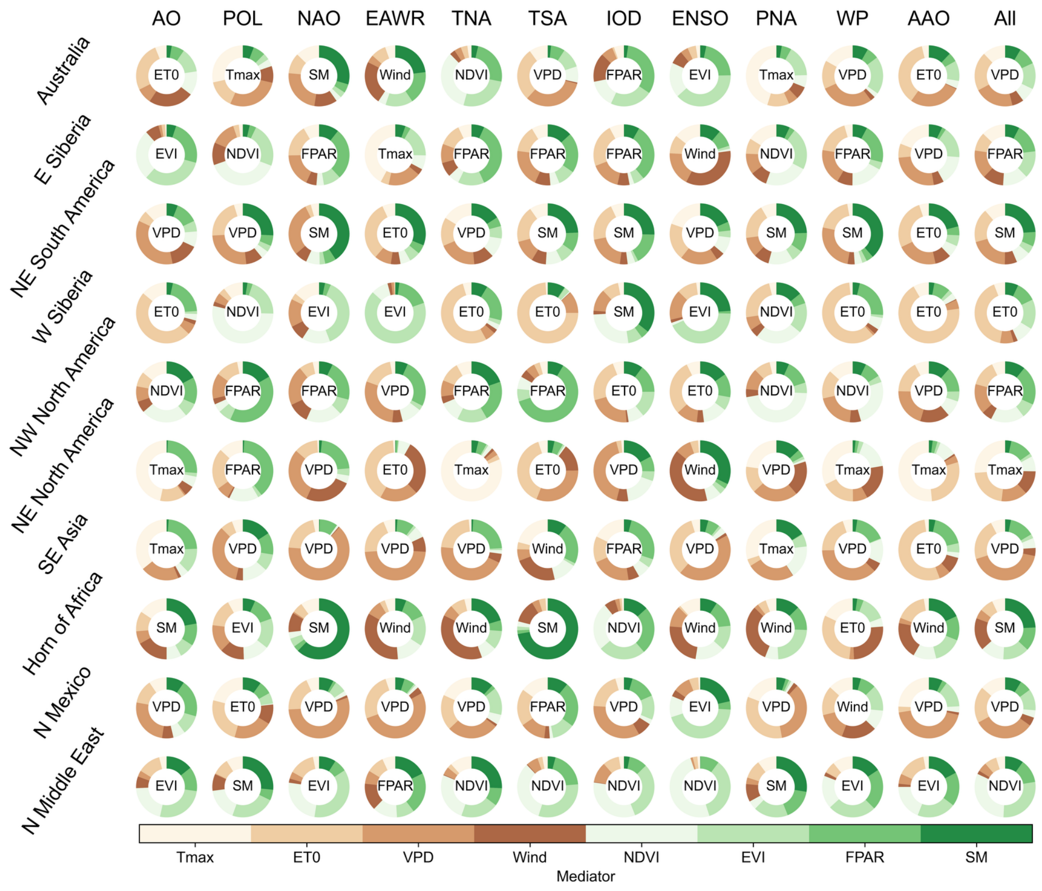

Figure S10. The mediators between teleconnection climate modes and burned area. Like Figure 5a, but for all hot spot regions and teleconnection climate modes.

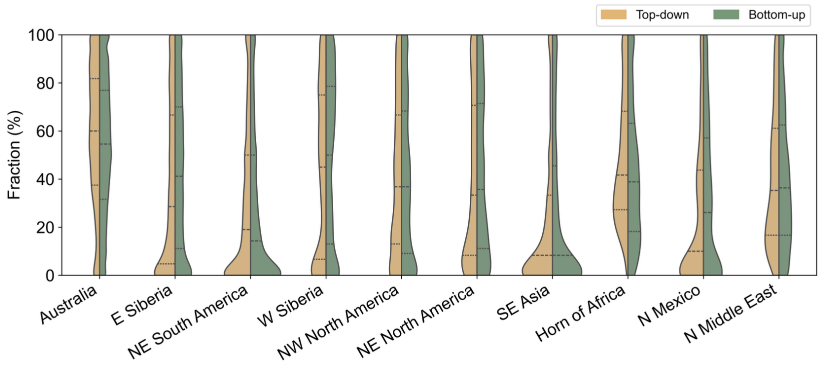

Figure S11. Distribution of time lags between top-down and bottom-up mediators and burned area. The fractions were calculated by dividing the time lags of mediators by the time lags of teleconnection climate modes. By calculating the fractions, we normalized the time lags for comparison across mediator groups, considering that teleconnection climate modes and mediators have varied time lags. In the violin plot, dashed lines indicate the upper quartile, median, and lower quartile, respectively.

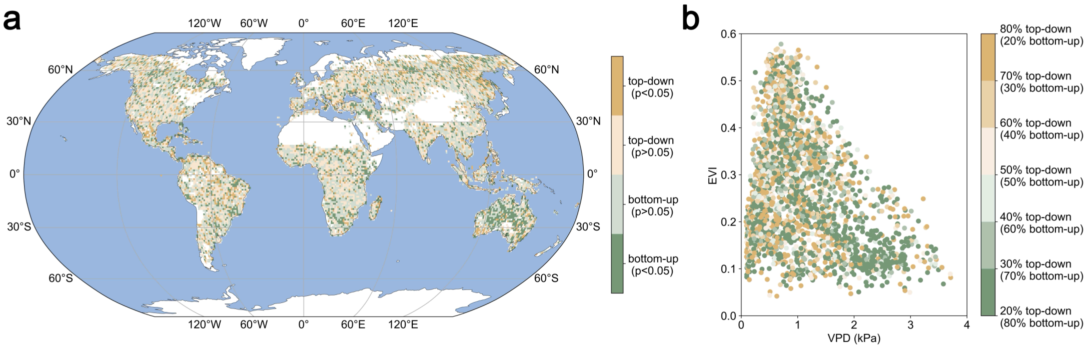

Figure S12. Comparison of the dominant mediator group according to group sum. (a) The dominant mediator group in linking the teleconnection climate mode (TCM) and the burned area (BA). In each pixel, the dominant group is defined as the group with the highest sum pathway contributions from all four precursors in that group. The pixels in dark (light) yellow and green indicate where the BA is predictable (not predictable), based on a significance threshold of 0.05. (b) For all the pixels where BA is predictable, the dominance of top-down and bottom-up mediator groups with vapor pressure deficit (VPD) and enhanced vegetation index (EVI). The dominance fraction is calculated by dividing the sum of pathway contributions of the top-down group by the sum of contributions of the two groups.

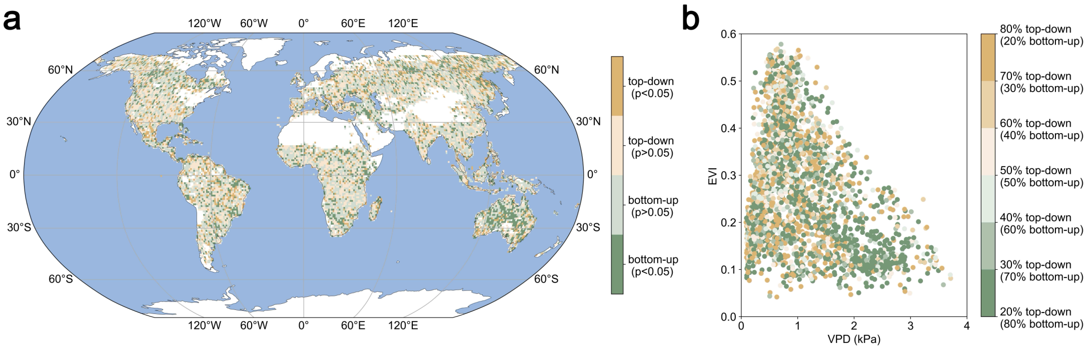

Figure S13. Comparison of the dominant mediator group according to the group maximum. Like Figure S12, but for the comparison between the most important mediator in each group.

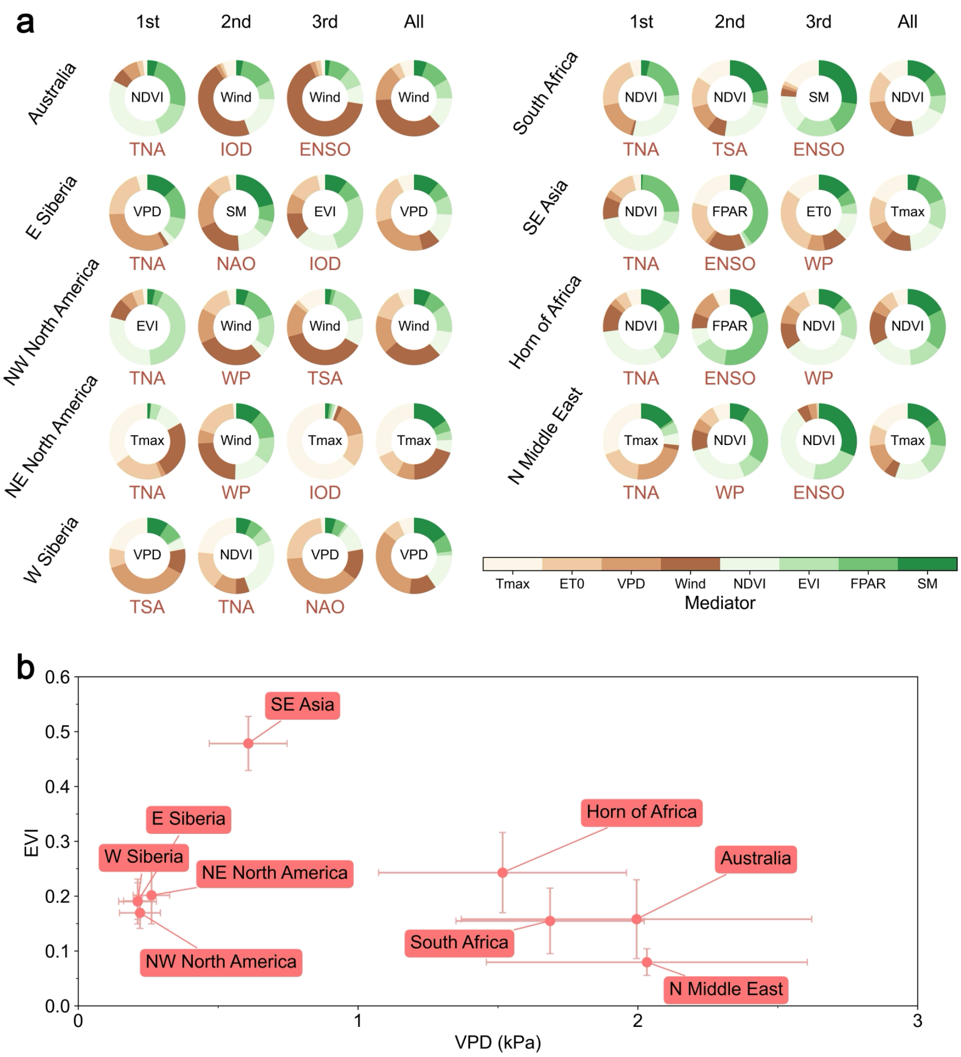

Figure S14. The mediators between teleconnection climate modes and fire radiative power. Like Figure 5, but for fire radiative power.
